# Common Variants at 9p21 and 8q22 Are Associated with Increased Susceptibility to Optic Nerve Degeneration in Glaucoma

**DOI:** 10.1371/journal.pgen.1002654

**Published:** 2012-04-26

**Authors:** Janey L. Wiggs, Brian L. Yaspan, Michael A. Hauser, Jae H. Kang, R. Rand Allingham, Lana M. Olson, Wael Abdrabou, Bao J. Fan, Dan Y. Wang, Wendy Brodeur, Donald L. Budenz, Joseph Caprioli, Andrew Crenshaw, Kristy Crooks, Elizabeth DelBono, Kimberly F. Doheny, David S. Friedman, Douglas Gaasterland, Terry Gaasterland, Cathy Laurie, Richard K. Lee, Paul R. Lichter, Stephanie Loomis, Yutao Liu, Felipe A. Medeiros, Cathy McCarty, Daniel Mirel, Sayoko E. Moroi, David C. Musch, Anthony Realini, Frank W. Rozsa, Joel S. Schuman, Kathleen Scott, Kuldev Singh, Joshua D. Stein, Edward H. Trager, Paul VanVeldhuisen, Douglas Vollrath, Gadi Wollstein, Sachiko Yoneyama, Kang Zhang, Robert N. Weinreb, Jason Ernst, Manolis Kellis, Tomohiro Masuda, Don Zack, Julia E. Richards, Margaret Pericak-Vance, Louis R. Pasquale, Jonathan L. Haines

**Affiliations:** 1 Department of Ophthalmology, Harvard Medical School, Massachusetts Eye and Ear Infirmary, Boston, Massachusetts, United States of America; 2 Vanderbilt University School of Medicine, Center for Human Genetics Research, Nashville, Tennessee, United States of America; 3 Duke University School of Medicine, Durham, North Carolina, United States of America; 4 Department of Medicine and Channing Laboratory, Brigham and Women's Hospital, Boston, Massachusetts, United States of America; 5 Broad Institute of Harvard and Massachusetts Institute of Technology, Boston, Massachusetts, United States of America; 6 Department of Ophthalmology, University of North Carolina School of Medicine, Durham, North Carolina, United States of America; 7 Department of Ophthalmology, University of California Los Angeles, Los Angeles, California, United States of America; 8 Center for Inherited Disease Research, Johns Hopkins School of Medicine, Baltimore, Maryland, United States of America; 9 Department of Ophthalmology, Johns Hopkins University School of Medicine, Baltimore, Maryland, United States of America; 10 Eye Doctors of Washington D.C., Washington D.C., United States of America; 11 Institute for Genomic Medicine and Marine Biology Research Division, University of California San Diego, San Diego, California, United States of America; 12 Department of Biostatistics, University of Washington, Seattle, Washington, United States of America; 13 Department of Ophthalmology, University of Miami, Miami, Florida, United States of America; 14 Department of Ophthalmology and Visual Sciences, University of Michigan, Ann Arbor, Michigan, United States of America; 15 Department of Ophthalmology, University of California San Diego, San Diego, California, United States of America; 16 Essentia Institute of Rural Health, Duluth, Minnesota, United States of America; 17 Department of Epidemiology, University of Michigan, Ann Arbor, Michigan, United States of America; 18 Department of Ophthalmology, West Virginia University School of Medicine, Morgantown, West Virginia, United States of America; 19 Department of Ophthalmology, University of Pittsburgh, Pittsburgh, Pennsylvania, United States of America; 20 Department of Ophthalmology, Stanford University, Palo Alto, California, United States of America; 21 The EMMES Corporation, Rockville, Maryland, United States of America; 22 Department of Genetics, Stanford University, Palo Alto, California, United States of America; 23 Computer Science and Artificial Intelligence Laboratory, Massachusetts Institute of Technology, Cambridge, Massachusetts, United States of America; 24 Department of Human Genetics, University of Miami School of Medicine, Miami, Florida, United States of America; Stanford University School of Medicine, United States of America

## Abstract

Optic nerve degeneration caused by glaucoma is a leading cause of blindness worldwide. Patients affected by the normal-pressure form of glaucoma are more likely to harbor risk alleles for glaucoma-related optic nerve disease. We have performed a meta-analysis of two independent genome-wide association studies for primary open angle glaucoma (POAG) followed by a normal-pressure glaucoma (NPG, defined by intraocular pressure (IOP) less than 22 mmHg) subgroup analysis. The single-nucleotide polymorphisms that showed the most significant associations were tested for association with a second form of glaucoma, exfoliation-syndrome glaucoma. The overall meta-analysis of the GLAUGEN and NEIGHBOR dataset results (3,146 cases and 3,487 controls) identified significant associations between two loci and POAG: the *CDKN2BAS* region on 9p21 (rs2157719 [G], OR = 0.69 [95%CI 0.63–0.75], p = 1.86×10^−18^), and the *SIX1/SIX6* region on chromosome 14q23 (rs10483727 [A], OR = 1.32 [95%CI 1.21–1.43], p = 3.87×10^−11^). In sub-group analysis two loci were significantly associated with NPG: 9p21 containing the *CDKN2BAS* gene (rs2157719 [G], OR = 0.58 [95% CI 0.50–0.67], p = 1.17×10^−12^) and a probable regulatory region on 8q22 (rs284489 [G], OR = 0.62 [95% CI 0.53–0.72], p = 8.88×10^−10^). Both NPG loci were also nominally associated with a second type of glaucoma, exfoliation syndrome glaucoma (rs2157719 [G], OR = 0.59 [95% CI 0.41–0.87], p = 0.004 and rs284489 [G], OR = 0.76 [95% CI 0.54–1.06], p = 0.021), suggesting that these loci might contribute more generally to optic nerve degeneration in glaucoma. Because both loci influence transforming growth factor beta (TGF-beta) signaling, we performed a genomic pathway analysis that showed an association between the TGF-beta pathway and NPG (permuted p = 0.009). These results suggest that neuro-protective therapies targeting TGF-beta signaling could be effective for multiple forms of glaucoma.

## Author Summary

Loss of vision from glaucoma, a common cause of blindness worldwide, is due to irreversible damage to the optic nerve. Current therapies cannot prevent glaucoma-related optic nerve disease and very little is known about the underlying responsible molecular events. Glaucoma patients affected by the “normal-pressure” subtype of glaucoma (NPG) have increased susceptibility to optic nerve degeneration. Although NPG has a high heritability, common predisposing genetic variants have not been identified. Therefore, we performed a meta-analysis of two independent genome-wide association studies for a common form of glaucoma, primary open angle glaucoma (POAG), followed by NPG subgroup analysis. We found that SNPs in the *CDKN2BAS* gene region on 9p21 and a highly conserved region with a probable regulatory function on 8q22 were associated with NPG and with optic nerve disease in a second type of glaucoma, exfoliation glaucoma. Both genomic regions are predicted to influence TGF-beta activity, and using whole-genome data we showed that the TGF-beta signaling pathway overall is associated with NPG. These results reveal new insights into the molecular pathogenesis of optic nerve disease in glaucoma and are an important step toward the development of preventative and protective therapies.

## Introduction

Glaucoma is a leading cause of blindness worldwide [1]. Primary open angle glaucoma (POAG), the most common form of glaucoma in the Western world, is an age-related, complex disease characterized by progressive irreversible degeneration of the optic nerve due to apoptotic retinal ganglion cell death [2]. In addition to age, epidemiologic studies have revealed multiple risk factors for the condition including elevated intraocular pressure (IOP), African-American race, family history and low ocular perfusion pressure [3]–[5]. Of these, elevated intraocular pressure (IOP) is the only treatable risk factor; however, many individuals have IOP elevation without optic nerve disease [6], and at least 33% of affected individuals have progressive retinal ganglion cell loss despite IOP measurements in the normal range (less than 22 mmHg), a condition defined as normal-pressure glaucoma (NPG) [7]. Preventative or neuro-protective therapies for glaucoma are not yet available and little is known about the molecular events that influence susceptibility to glaucomatous optic nerve degeneration.

POAG is genetically complex [8]. Linkage studies have identified over 20 genomic regions likely to contain POAG-related genes [9]. Importantly, genes that influence POAG risk overall may specifically contribute to separate independent biological processes affecting the disease outcome including regulation of IOP and retinal ganglion cell physiology. Individuals with elevated IOP without optic nerve disease may only carry genetic variants that influence IOP regulation, while individuals with NPG may primarily carry genetic variants that predispose to retinal ganglion cell death as the nerve degenerates in these patients without the added stress of elevated IOP.

Recent genome-wide association studies (GWAS) have identified several genetic risk factors for POAG overall, including single-nucleotide polymorphisms (SNPs) located in the CAV1/CAV2 intergenic region [10]–[11], and in the genomic regions containing the *TMCO1* and *CDKN2BAS* genes [12] in a study of glaucoma patients with advanced optic nerve disease. Linkage studies have identified two genes that contribute to rare familial forms of NPG, *OPTN* (optineurin) [13] and *TBK1* (TANK-binding kinase) [14], and an intronic SNP in *SRBD1* (S1 RNA binding domain) has been associated with NPG in a GWAS of 355 Japanese cases [15]. Genome-wide association studies have not yet identified genes commonly associated with normal-pressure glaucoma or with optic nerve disease in glaucoma. The identification of genes that influence glaucoma-related optic nerve degeneration is an important step toward the development of neuro-protective therapies that could substantially reduce the morbidity caused by this common disease, and such therapies could be relevant to optic nerve degeneration occurring in many chronic forms of glaucoma.

To identify genes that predispose to glaucomatous optic nerve disease, we completed two GWAS for POAG: the GLAUGEN (Glaucoma Genes and Environment) GWAS that is part of the GENEVA (GENEVA Genes Environment Association) studies [16] and the NEIGHBOR (NEI Glaucoma Human genetics collaBORation) GWAS [17]. We then performed a meta-analysis as well as normal-pressure and high-pressure subgroup analyses of the combined dataset. To determine if the observed associations were specific to POAG or could generalize more broadly to optic nerve disease in other forms of glaucoma we selected the lead SNPs showing significant association in the normal-pressure glaucoma subgroup analysis, and tested them for association with glaucoma in a population of unrelated individuals with exfoliation syndrome glaucoma.

## Results

### Genome-wide association studies and meta-analyses

After data cleaning, 976 cases and 1,140 controls collected from three study sites [Nurses' Health Study, Health Professionals Follow-up Study and the Genetic Etiologies of Primary-Open angle Glaucoma Study (GEP)] were analyzed for the GLAUGEN study, and 2,170 cases and 2,347 controls collected from 12 sites (Table S1) were analyzed for the NEIGHBOR study. All cases and controls for both studies were residents of the continental United States and were of mainly European ancestry, which was confirmed by principal component analysis. The general characteristics for the cases and controls are shown in Table 1. For POAG overall in the GLAUGEN GWAS, there were no SNPs with p-values that reached the genome-wide significance level of p = 5×10^−8^ (Figure S1 and Figure S2).

**Table 1:** Features of cases and controls.

	Number		Mean Age (years)*		% Female	
Dataset	Cases	Controls	Cases	Controls	Cases	Controls
GLAUGEN	976	1140	63.6	65.5	60%	58%
NEIGHBOR	2170	2347	66.4	68.0	53%	54%
Meta-analysis (all)	3146	3487	65.2	67.5	54%	57%
Meta-analysis (NPG)	720	3443	61.2	67.5	58%	57%
Meta-analysis (HPG)	1669	3487	65.3	67.6	52%	57%

**GLAUGEN = Glau**coma Genes and **En**vironment study.

**NEIGHBOR = NEI** **G**laucoma **H**uman genetics colla**BOR**ation study.

**NPG = **Normal-pressure glaucoma defined as IOP <22 mm Hg at diagnosis.

**HPG = **High-pressure glaucoma defined as IOP ≥22 mm Hg at diagnosis.

*: The mean for each group is listed.

In the NEIGHBOR overall POAG GWAS, there were seventeen SNPs that reached the genome-wide significance level of p = 5×10^−8^. Sixteen of the significant SNPs were found in the *CDKN2BAS* gene region on chromosome 9p21.3 (top SNP rs4977756 (OR = 0.66 [95% CI 0.59–0.73], p value = 7.4×10^−16^). The remaining significant SNP rs10483727 was located in the SIX1/SIX6 region on chromosome 14 (OR = 1.32 [95% CI 1.20–1.46], p value = 3.1×10^−8^). (Figure S3 and Figure S4; Table S2).

Using METAL [18], we conducted a meta-analysis of the GLAUGEN and NEIGHBOR datasets that included a total of 3,146 cases and 3,487 controls and found nineteen SNPs that achieved genome-wide significance for POAG overall (Figure S5): seventeen of them in the *CDKN2BAS* region, with the most significant SNP being rs2157719 (OR = 0.69 [95%CI 0.63–0.75], p = 1.86×10^−18^, and two SNPs in the *SIX1/SIX6* region on chromosome 14q23 (most significant SNP: rs10483727, OR = 1.32 [95%CI 1.21–1.43], p = 3.87×10^−11^) (Table S3). A number of genomic regions, including the *CDKN2BAS* and *SIX1/SIX6* regions have been previously associated with optic nerve quantitative parameters (cup-to-disc ratio (CDR) and optic nerve area) [19]–[24]. Of these, only the *CDKN2BAS* and *SIX1/SIX6* regions were significantly associated with glaucoma in the meta-analysis, although several other previously identified gene regions demonstrated suggestive associations including *SALL1*, *LRP1B* and *SIRPA* (Table 2, Table S4).

**Table 2:** Glaucoma association results for SNPs in gene regions associated with quantitative optic nerve parameters optic nerve area and cup-to-disc ratio (CDR).

Optic nerve area								
Gene region	Reference	SNP*	P overall	OR overall	P HPG	OR HPG	P NPG	OR NPG
ATOH7	19,20,21	rs17231602:A	0.049	0.89	0.322	0.93	0.903	1.01
RFTN1	19	rs12487874:A	4.80E-03	1.15	0.307	1.07	5.03E-03	1.27
CDC7/TGFBR3	20, 21, 22	rs11165816:G	2.04E-03	0.79	0.064	0.84	0.014	0.73
SALL1	20	rs3743795:G	0.320	0.95	0.487	0.95	8.54E-04	0.74
CARD10	21	rs9607468:A	0.289	0.92	0.662	0.96	8.44E-03	0.69
ELP4-PAX6	23	rs677874:C	0.749	0.98	0.872	0.99	0.0126	0.80
LRP1B	22	rs2381190:C	8.90E-04	1.17	0.024	1.15	0.507	1.06

Optic nerve morphology (Cup-to-disc ratio)								
Gene region	Reference	SNP*	P overall	OR overall	P HPG	OR HPG	P NPG	OR HPG
CDKN2B	20	rs1063192:G	2.30E-17	1.43	3.21E-05	1.26	1.13E-11	1.68
SIX1/SIX6	20	rs10483727:A	3.87E-11	1.32	6.67E-07	1.31	8.66E-05	1.33
SCYL1/LTBP3	20	rs1787663:A	0.123	0.93	0.378	0.95	0.141	0.89
CHEK2	20	rs134173:A	8.84E-03	0.90	0.501	0.97	0.044	0.87
DCLK1	20	rs11839572:T	0.486	1.04	0.629	1.03	2.58E-03	1.31
SIRPA	22	rs6034909:G	0.031	0.89	6.27E-04	0.79	0.627	1.05
RNF24/PANK2	22	rs6037744:A	4.71E-03	1.12	0.027	1.13	0.081	1.13
RERE	22	rs953043:C	4.28E-03	0.88	0.033	0.88	0.117	0.89
IGFBP2	22	rs12999653:A	0.999	1.00	0.594	1.05	0.058	0.78
DCLK3	22	rs954493:A	0.124	1.08	0.492	1.05	0.026	1.21

*: The SNP in the gene region with the best evidence of association, either in the overall meta-analysis or in the HPG or NPG subgroups was selected for inclusion in this table. The effect allele is listed after the SNP number. Complete results are presented in Table S4. Abbreviations: POAG (primary open angle glaucoma); HPG (high-pressure glaucoma); NPG (normal-pressure glaucoma); OR (odds ratio).

### Normal-pressure and high-pressure subgroup analyses

**Figure 1:**
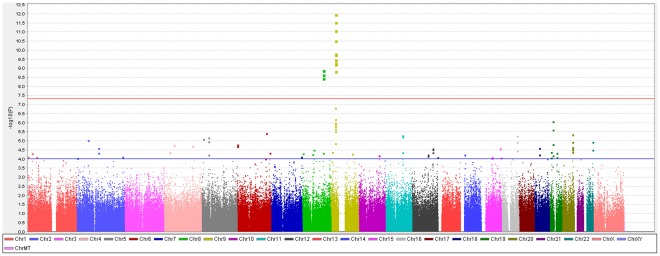
Genome-wide association results with normal pressure glaucoma (NPG) (IOP <22 mm Hg) in the GLAUGEN-NEIGHBOR meta-analysis (720 cases and 3487 controls). The red line identifies a p-value of 5×10^−8^. Covariates include: (NEIGHBOR) age, gender, study site and eigenvectors 1 and 2; (GLAUGEN) age, gender, study site, DNA extraction method, DNA specimen type and eigenvectors 1, 2 and 6.

**Figure 2:**
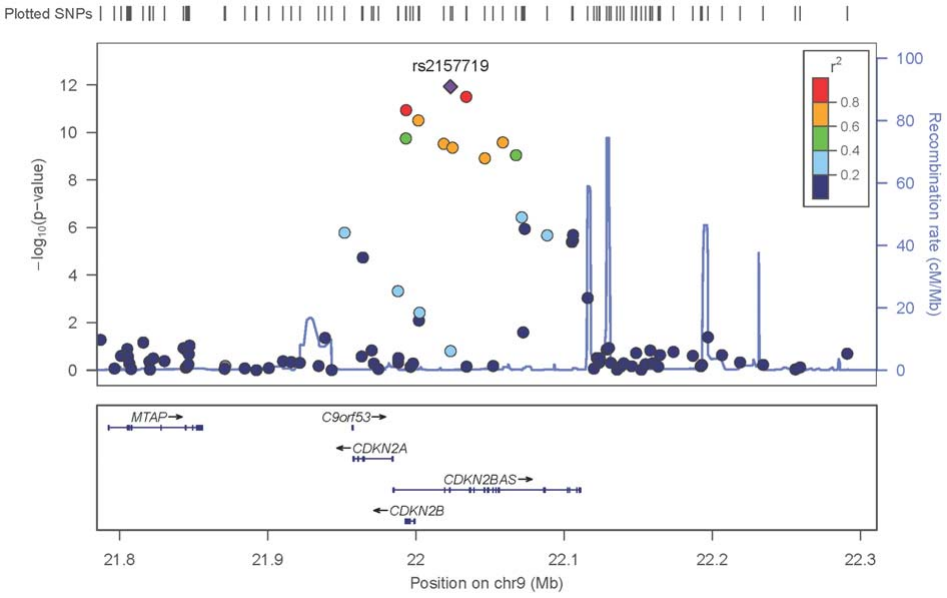
Meta-analysis results for SNPs associated with normal pressure glaucoma (NPG) (IOP <22 mmHg) located in the CDKN2BAS region on chromosome 9p21. The most significant SNP (rs2157719, p = 1.17×10^−12^) is indicated with a solid diamond.

**Figure 3:**
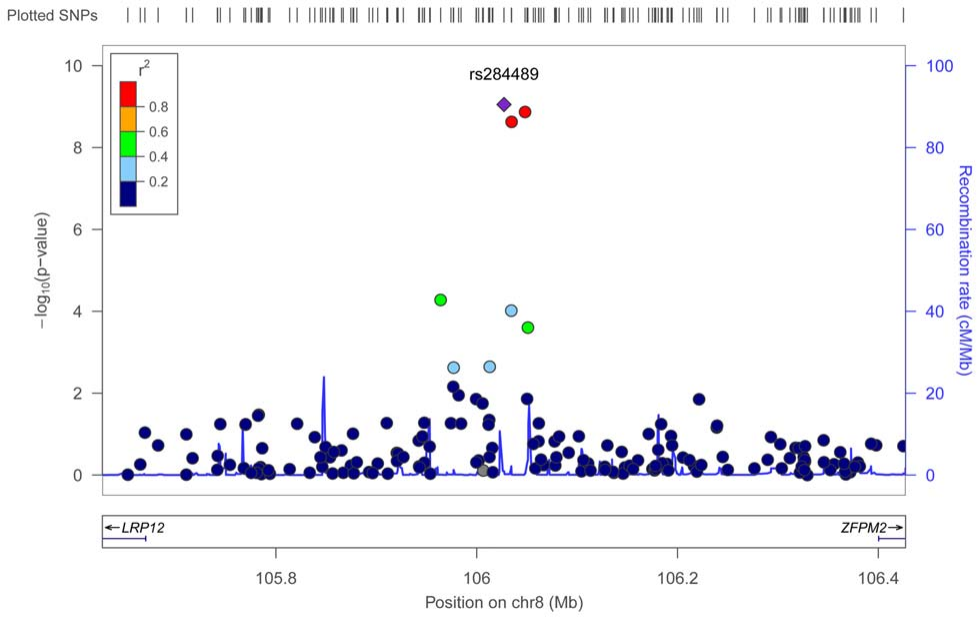
Meta-analysis results for SNPs associated with normal pressure glaucoma (NPG) (IOP <22 mm Hg) located in the 8q22 region. The most significant SNP (rs284489, p = 8.8×10^−10^) is indicated with a solid diamond.

To identify genetic risk factors primarily associated with optic nerve disease in glaucoma, we performed separate meta-analyses for NPG (720 cases) and high-pressure glaucoma (HPG, 1669 cases with IOP ≥22 mm Hg), (757 cases did not have untreated IOP data available). In the HPG meta-analysis, no SNPs, even those in the *CDKN2BAS* and *SIX1/SIX6* regions, reached genome-wide significance (Figure S6). In the NPG meta-analysis, SNPs in two regions reached genome-wide significance, (Figure 1, Table 3): thirteen SNPs in the *CDKN2BAS* region on 9p21 (most significant SNP was rs2157719, (OR = 0.58 [95% CI 0.50–0.67], p = 1.17×10^−12^) (Figure 2, Figure S7) and three SNPs in an evolutionarily conserved region on chromosome 8q22 (most significant SNP was rs284489, OR = 0.62 [95% CI 0.53–0.72], p = 8.88×10^−10^) (Figure 3, Figure S8).

**Table 3:** Association results for genome-wide significant SNPs for the Normal Pressure Glaucoma (NPG) analyses.

		Meta-analysis		NEIGHBOR				GLAUGEN			
SNP: Minor allele	Chr	P	OR (95% CI)	MAF		P	OR (95% CI)	MAF		P	OR (95% CI)
				Cases	Controls			Cases	Controls		
rs2157719:G	9	1.17E-12	0.58 (0.50–0.67)	0.27	0.42	1.70E-07	0.58 (0.47–0.71)	0.31	0.42	8.98E-07	0.58 (0.46–0.72)
rs1412829:G	9	3.12E-12	0.58 (0.50–0.68)	0.27	0.42	2.86E-07	0.58 (0.47–0.71)	0.30	0.41	1.48E-06	0.58 (0.46–0.72)
rs1063192:G	9	1.13E-11	0.60 (0.51–0.69)	0.28	0.43	2.71E-07	0.59 (0.48–0.72)	0.32	0.43	5.63E-06	0.60 (0.49–0.75)
rs573687:A	9	3.07E-11	0.58 (p.50–0.68)	0.24	0.38	1.38E-06	0.59 (0.47–0.73)	0.27	0.36	3.22E-06	0.58 (0.46–0.73)
rs3217992:A	9	1.75E-10	1.60 (1.38–1.84)	0.53	0.39	4.45E-06	1.56 (1.29–1.89)	0.47	0.39	5.90E-06	1.64 (1.32–2.02)
rs4977756:G	9	2.59E-10	0.61 (0.53–0.71)	0.26	0.40	7.31E-07	0.59 (0.48–0.73)	0.29	0.39	4.86E-05	0.64 (0.51–0.79)
rs7049105:A	9	2.96E-10	0.63 (0.55–0.73)	0.37	0.52	9.45E-06	0.65 (0.53–0.78)	0.41	0.49	4.81E-06	0.61 (0.50–0.76)
rs2151280:A	9	4.28E-10	0.64 (0.55–0.74)	0.38	0.52	2.22E-05	0.66 (0.55–0.80)	0.42	0.48	2.72E-06	0.61 (0.49–0.75)
rs284489:G	8	8.88E-10	0.62 (0.53–0.72)	0.29	0.34	3.65E-05	0.64 (0.52–0.79)	0.30	0.38	3.75E-06	0.59 (0.48–0.74)
rs1412832:G	9	8.90E-10	0.59 (0.50–0.70)	0.19	0.31	1.36E-06	0.56 (0.45–0.71)	0.21	0.31	1.48E-06	0.58 (0.47–0.72)
rs10120688:C	9	1.20E-09	0.64 (0.56–0.74)	0.37	0.50	2.67E-05	0.66 (0.55–0.80)	0.41	0.50	6.92E-06	0.62 (0.50–0.76)
rs1521774:G	8	1.35E-09	0.62 (0.53–0.72)	0.29	0.33	6.6E-05	0.65 (0.52–0.80)	0.30	0.37	3.04E-06	0.58 (0.47–0.74)
rs284495:G	8	2.35E-09	0.63 (0.53–0.72)	0.29	0.34	6.74E-05	0.65 (0.53–0.80)	0.30	0.37	5.50E-06	0.60 (0.48–0.75)

**Chr = **chromosome; **MAF = **minor allele frequency; **OR = **odds ratio; **CI = **confidence interval.

### Single–SNP associations

#### Chromosome 9p21 region

After imputation 90 SNPs in the 9p21 region were significantly associated with NPG (Table S5), although conditional analysis showed that none of these were independent of rs2157719, the most significantly associated SNP in the meta-analysis. The minor allele for rs2157719 showed a protective effect, and is located within the genomic region coding for the noncoding RNA, *CDKN2BAS*, also known as ANRIL, which regulates expression of *CDKN2B* as well as other genes [25]. rs2157719 falls within a DNase I hypersensitivity site [26] that has a significant signal in a variety of cell types [27] (Figure S9). Targeted re-sequencing of the *CDKN2BAS* gene in a subset of 11 cases carrying a protective haplotype that includes rs2157719 (Table S6) identified two rare variants (rs7341786 [A]; rs7341791 [A]) in 8 of the 11 individuals. After imputation we found that these variants are significantly associated with glaucoma (p = 2.73×10^−9^, OR = 0.75; p = 3.09×10^−9^, OR = 0.75 respectively). These SNPs have been previously shown to affect RNA splicing [28], suggesting that altering *CDKN2BAS* expression may contribute to glaucoma.

#### Chromosome 8q22 region

The three SNPs located in the 8q22 region that were significantly associated with NPG in the subgroup analysis are in strong linkage disequilibrium (r^2^>0.8) (Figure S8), and imputation followed by conditional analysis did not reveal additional independent associations. The genomic region containing the associated SNPs includes highly conserved DNA sequences and several DNaseI hypersensitivity sites [26], [27] (Figure S10). An imputed SNP (rs284492) in strong LD with the lead SNP (rs284489) falls within or proximal to DNaseI hypersensitivity sites with significant activity in choroid plexus epithelial cells, as well as two ocular tissues (nonpigmented ciliary epithelial cells, and iris pigment epithelial cells) (Figure S10, Table S5). Haplotypes significantly associated with NPG (Table S7) include these DNaseI hypersensitivity sites. This putative regulatory region is located within a 630 Kb gene desert flanked by *LRP12* (Low-density lipoprotein receptor related protein 12) and *ZFPM2* (Zinc finger protein multitype 2) and could influence expression of either of these genes.

### Ocular expression of chromosome 8 genes LRP12 and ZFPM2

Using RT-PCR, we found that both *LRP12* and *ZFPM2* are expressed in the human optic nerve, as well as in other ocular tissues relevant to glaucoma (Figure S11). In mouse experiments using laser microdissection to selectively study specific layers of the retina, both *LRP12* and *ZFPM2* were expressed in the retinal ganglion cell and inner nuclear layers (Figure S11), making them good candidates for optic nerve susceptibility genes.

### DNA sequencing of LRP12 and ZFPM2

We performed targeted resequencing of the LRP12 and ZFPM2 genes in 16 NPG patients and also analyzed the exomes of 50 POAG cases and 18 controls for DNA sequence variants in both genes (Table S8). None of the variants identified are expected to significantly affect gene expression or protein function.

### Association of 9p21 and 8q22 SNPs with exfoliation glaucoma

To determine if SNPs associated with NPG were associated with optic nerve degeneration in other types of glaucoma, we examined the association of the top SNPs in both the 9p21 and 8q22 genomic regions with optic nerve degeneration in exfoliation glaucoma, another form of open-angle glaucoma. Exfoliation syndrome, a condition predisposing to elevated intraocular pressure, is associated with genetic variants in *LOXL1* [29], however, these variants appear to contribute to the development of the exfoliation syndrome and not to optic nerve disease in POAG [30]. We found that rs2157719 in 9p21 was significantly associated with exfoliation glaucoma (Table 4), and rs284489 in 8q22 was also nominally associated with the same directionality of the association observed for NPG.

**Table 4:** Association results for exfoliation glaucoma and 9p21 and 8q22 SNPs.

		Exfoliation All				Exfoliation glaucoma				Exfoliation only			
SNP: Minor allele	Chr	MAF		P*	OR** (95%CI)	MAF		P*	OR** (95%CI)	MAF		P*	OR** (95%CI)
		Cases N = 196	Controls N = 344			Cases N = 104	Controls N = 344			Cases N = 92	Controls N = 344		
rs2157719:G	9	0.36	0.42	0.032	0.71 (0.52–0.95)	0.31	0.42	0.004	0.59 (0.41–0.87)	0.40	0.42	0.54	0.79 (0.52–1.20)
rs284489:G	8	0.35	0.41	0.066	0.84 (0.65–1.10)	0.31	0.41	0.021	0.76 (0.54–1.06)	0.43	0.41	0.610	1.04 (0.71–1.52)

*: p-values were calculated using chi-square and the odds ratio (OR).

**: was calculated using a logistic regression model that includes age as a covariate. Exfoliation All includes all exfoliation cases, with and without glaucoma.

### TGF–beta pathway analysis

Both the 9p21 and 8q22 regions could contribute to TGF-beta signaling. The 9p21 SNPs could effect expression of *CDKN2BAS*, a noncoding RNA that influences expression of *CDKN2B*, a member of the TGF-beta signaling pathway [31]. Using the SCAN database [32], we found that the three 8q22 SNPs associated with NPG (rs284489, rs284495 and rs1521774) influence the expression of *TSC22 (TGF-beta stimulated clone 22)*, which also modulates signaling by TGF-beta [33]. Using the PARIS (Pathway Analysis by Randomization Incorporating Structure) algorithm [34], we found that the TGF-beta pathway (KEGG, hsa04350) Kyoto Encyclopedia of Genes and Genomes) overall was associated with NPG in our combined dataset (permuted p = 0.009).

## Discussion

Using two large POAG case-control datasets, we have completed a meta-analysis for POAG followed by subgroup analyses for NPG and HPG. We have identified two genomic regions that are associated with NPG and showed that the lead SNP in each region is also associated with optic nerve disease in a second type of open-angle glaucoma (exfoliation syndrome related glaucoma).

The first region includes the *CDKN2BAS* gene on chromosome 9p21, previously associated with cup-to-disc ratio (CDR) an optic nerve quantitative parameter, as well as POAG in candidate gene studies [20], [35], [36] and more recently in a GWAS using a sample of severely affected POAG patients [12]. In this study, we show that this gene region is associated with NPG suggesting that *CDKN2BAS* contributes to optic nerve degeneration in glaucoma. *CDKN2BAS* codes for an antisense RNA that regulates the expression of *CDKN2B*, which is an inhibitor of cyclin-dependent kinase 4 (CDK4), a protein kinase that has a pivotal role in cell cycle progression [37]. The minor alleles of the NPG associated SNPs are protective, suggesting that these SNPs, or other variants in linkage disequilibrium with these SNPs, could influence *CDKN2BAS* and *CDKN2B* expression with corresponding changes in cyclin-dependent kinase activity that could result in retinal ganglion cell apoptosis. *CDKN2BAS* expression can also be influenced by interferon alpha [38]. As inflammation and autoimmunity may be factors contributing to glaucoma pathogenesis [39], the regulation of *CDKN2BAS* expression by interferon could suggest a direct role for *CDKN2BAS* in glaucoma pathogenesis. *CDKN2BAS* also regulates the expression of *CDKN2A*, a gene previously shown to be down-regulated in other neurodegenerative disorders, including Alzheimer's disease, suggesting that regulation of *CDKN2A* expression by *CDKN2BAS* could also contribute to degeneration of the optic nerve in glaucoma [40].

The second region associated with NPG in this study is an evolutionarily conserved DNA segment on chromosome 8q22 with predicted regulatory function. This region partially overlaps with a POAG locus defined by a linkage study of a single large family with low intraocular pressure (GLC1D) [41], and a second linkage study using multiple affected families stratified by age of disease onset [42]. The conserved region falls within a 630 Kb gene desert flanked by the *LRP12* and *ZFPM2* genes. *ZFPM2*, also known as *FOG2*, codes for a zinc finger protein that appears to contribute to cardiovascular development, and may have a role in ocular development [43]. ZFPM2 is expressed in the eye, and expression was observed to increase after injury to the optic nerve [44]. The *LRP12* gene is a member of the *LRP* (low-lipoprotein receptor) gene family, with several members previously implicated in glaucoma. LRP1 is decreased in the optic neuropathy associated with Alzheimer's disease [45], *LRP1B* is associated with optic nerve area [22], a knock-out of *LRP2* causes a glaucoma-like phenotype in zebrafish [46], *LRP4* expression is increased in response to retinal ischemia [47], and expression of *LRP10* and *LRP11* is up-regulated in a rat model of glaucoma [48], [49]. Importantly, LRP12, previously known as ST7, is a receptor for SMAD4, a major regulator of the TGF-beta signaling pathway [50]. Interestingly, SMAD4 influences activity of TSC22 [51].

The 8q22 genomic region associated with NPG contains putative regulatory sites that appear to be active in two cell types that could contribute to glaucoma-related optic nerve disease, choroid plexus epithelial cells and non-pigmented ciliary body epithelial cells. The choroid plexus is responsible for formation of cerebral spinal fluid, and recent studies have suggested that low cerebral spinal fluid pressure, which could be caused by decreased formation of cerebral spinal fluid, may create a deleterious gradient across the lamina cribrosa in NPG mimicking a similar gradient induced by higher IOP in HPG [52], [53]. The 8q22 regulatory region is also active in non-pigmented ciliary body epithelial cells, which affect ocular intraocular pressure through the production of aqueous humor (a fluid that is similar in composition to cerebral spinal fluid).

Both genomic regions identified in this study could contribute to regulation of TGF-beta signaling and our pathway analysis provides additional support for a role for TGF-beta signaling in glaucomatous optic nerve disease and retinal ganglion cell death. TGF-beta and other members of the TGF-beta signaling pathway have been previously implicated in glaucoma [54], [55], and genes participating in TGF-beta signaling are expressed in ocular structures that are involved in glaucoma, including the anatomic structures regulating IOP and the optic nerve [56]–[58]. TGF-beta1 is up-regulated in optic nerve tissue in an animal model of glaucoma [59], and mutations in *LTBP2* (latent transforming growth factor beta binding protein 2) cause a rare autosomal recessive form of congenital glaucoma [60]. Collectively, these results suggest that therapies directed toward the regulation of TGF-beta signaling could protect the optic nerve from degeneration in glaucoma. The discovery of POAG genetic risk factors, especially the factors that predispose to glaucoma-related optic neuropathy, is a critical first step toward understanding the pathophysiology of POAG and the development of gene-based screening tests and neuro-protective therapies for this common blinding disease.

## Methods

### Subjects

The institutional review boards of the Massachusetts Eye and Ear Infirmary, Harvard School of Public Health, the Brigham and Women's Hospital, University of Pittsburgh, Johns Hopkins University, Duke University, University of West Virginia, University of Miami, University of Michigan, Stanford University, Marshfield Clinic, and the University of California, San Diego. approved this study.

#### Clinical definitions

POAG cases were defined as individuals for whom reliable visual field (VF) tests show characteristic VF defects consistent with glaucomatous optic neuropathy. Individuals were classified as affected if the VF defects were reproduced on a subsequent test or if a single qualifying VF was accompanied by a cup-disc ratio (CDR) of 0.7 or more in at least one eye. The examination of the ocular anterior segment did not show signs of secondary causes for elevated IOP such as exfoliation syndrome or pigment dispersion syndrome and the filtration structures were deemed to be open based on clinical measures. Elevation of IOP was not a criterion for inclusion; however, 67% of cases did have a history of elevated IOP (≥22 mm Hg) measured in a clinical setting (typically between the hours of 8AM and 5PM) and were classified as high-pressure glaucoma (HPG). Cases with IOP <22 mm Hg measured in the clinic at the time of study enrollment (without treatment) were classified as normal-pressure glaucoma (NPG). Cases undergoing IOP-lowering therapy at the time of enrollment were included in the HPG group if they had a documented history of IOP >22 prior to treatment and cases undergoing IOP-lowering therapy at the time of enrollment were included in the NPG if they did not have recorded pressures >22 mmHg before treatment. As glaucoma patients are long-term patients with several clinic visits each year, for most of the glaucoma cases in this study the IOP measurements were made at least twice on multiple occasions. Exfoliation glaucoma cases had evidence of characteristic fibrillar deposits as previously described [61] in addition to glaucoma as defined above. Controls had normal optic nerves (cup-disc ratios ≤0.6) and normal intraocular pressure (≤21 mm Hg).

#### Dataset descriptions

The GLAUGEN dataset included 976 cases and 1,140 controls drawn from three different studies: the Genetic Etiologies of Primary-Open angle Glaucoma (GEP), the Nurses' Health Study (NHS) and the Health Professionals Follow-up study (HPFS). The GEP is a clinic-based case-control set, and the NHS and HPFS are case-control sets nested within population-based studies. Additional information about the case identification and control selection process in GLAUGEN can be found at The Primary Open-Angle Glaucoma Genes and Environment (GLAUGEN) Study. Study Accession: phs000308.v1.p1. www.ncbi.nlm.nih.gov/projects/gap. December 21, 2010. The majority of DNA samples were prepared using Qiagen extraction kits (Invitrogen). More than half of the samples were derived from buccal cells, and we previously demonstrated the feasibility of genotyping buccal cell DNA on the Illumina 660W Quad platform [62].

Cases and controls for the NEIGHBOR study were collected from 12 sites (Table S1). Additional information about the NEIGHBOR consortium can be found in reference 17.

### Genome-wide association studies

#### Genotyping and quality control

Genotyping for the GLAUGEN and NEIGHBOR samples was completed using the Illumina Human660W_Quad_v1 array. (Illumina, San Diego, CA) at the Broad Institute (GLAUGEN) and the Center for Inherited Diseases Research (CIDR) (NEIGHBOR). At both of the genotyping centers, samples were plated to allow equal representation of cases and controls per plate from each study site in order to minimize batch effects.

For the GLAUGEN samples genotyping calls were generated using Illumina's BeadStudio, GenomeStudio and Autocall software along with genotype cluster definitions based on study samples. SNPs with a GenTrain score <0.636, cluster separation score <0.4 and call rate <97% were considered technical failures at the genotyping center and were automatically deleted before release for further quality control. Data was released for 2,241 study samples (95% of attempted samples). Subsequent data quality control measures consisted of identifying and removing samples with gender misidentification, unexpected duplicates and unexpected relatedness. Analysis of connectivity removed samples that appeared to be related to other samples and/or suggestive of contamination. Any SNP with missing call rate >2% or with Hardy Weinberg p-value<10^−4^ in the control population was excluded. Logistic regression analysis indicated that study site (GEP, NHS or HPFS), DNA source (blood or cheek cell), and DNA extraction method (DNAzol, Qiagen or GENTRA) were independent predictors of genotyping call rate for the GLAUGEN samples. Hence, these variables along with age and gender were used in the logistic regression models.

For the NEIGHBOR samples data from CIDR was released for 5,155 study samples (97% of attempted samples). Study samples, including 117 study duplicates, were plated and genotyped together with 227 HapMap controls (208 CEU; 11 YRI, 4 JPT, 4 CHB). Genotyping was performed using Illumina Human660W-Quadv1_C BeadChips (Illumina, San Diego, CA, USA) and the Illumina Infinium II assay protocol [63]. Allele cluster definitions for each SNP were determined using Illumina GenomeStudio Genotyping Module version 1.7.4, GenTrain version 1.0 and the combined intensity data from 99.9% of the samples. The resulting cluster definitions were used on all samples. Genotypes were not called if the quality threshold (Gencall score) was below 0.15. Genotypes were released by CIDR for 557,029 SNPs (99.58% of attempted). Genotypes were not released for SNPs that had call rates less than 85%, more than 1 HapMap replicate error, cluster separation less than 0.2, more than a 3% (autosomal) or 2.2% (X chromosome) difference in call rate between genders, more than 0.4% (X chromosome) male heterozygosity, or more than a 8% (autosomal) difference in AB frequency. XY, Y and mitochondrial SNPs were manually reviewed and clusters adjusted or genotypes dropped as appropriate. Intensity data was released for all attempted SNPs. The mean non-Y SNP call rate and mean sample call rate was 99.9% for the released CIDR dataset. Study duplicate reproducibility was 99.99%. After applying quality control filters, 495,132 SNPs were analyzed in 976 cases and 1140 controls for GLAUGEN and 523,528 SNPs were analyzed in 2170 cases and 2347 controls for NEIGHBOR.

#### Association analysis

Logistic regression to assess the association between individual SNPs and POAG was done using PLINK v1.07 [64]. For GLAUGEN, the logistic regression model included age, gender, study site, DNA source, DNA extraction method and 3 eigenvectors (EV 1, 2 and 6). For NEIGHBOR, the logistic regression model included age, gender, study site and 2 eigenvectors (EV1 and 2). Quantile-quantile plots (Figures S1 and S3) were used to estimate genomic inflation factors which were 1.009 for GLAUGEN and 1.034 for NEIGHBOR.

### Meta-analysis

Combined meta-analysis of the GLAUGEN and NEIGHBOR datasets was done using the METAL [18] software package. We analyzed each study using logistic regression as described previously. Then, we combined the results using the inverse weighted variance method based on the regression coefficients and standard errors estimated from each study as implemented in the program METAL [18]. The GENOMICCONTROL option was set to ON to adjust for genomic inflation differences between the studies.

### Haplotype analysis

Haplotype analysis (logistic regression) was performed with PLINK 1.07 [64] using sliding windows of 2–6 SNPs across the associated regions. Covariates in the model were the same as for the single-allele analyses.

### Pathway analysis

Pathway analysis was done using the Pathway Analysis by Randomization Incorporating Structure (PARIS) pathway analysis software package [34]. Genes comprising the transforming growth factor beta (TGF-beta) pathway were identified using the KEGG database (hsa:04350). SNPs were considered to reside in a pathway gene if the SNP fell within the ENSEMBLE genomic interval+/−50 kb to either side of the gene. If the overlap included another gene, the overlapping SNP(s) were counted once. A single-allele p-value of <0.05 was considered to be nominally significant and included in the PARIS analysis.

### Imputation

Focused regional imputation was used to infer genotypes for SNPs not directly genotyped using MACH 1.0 [65], [66] to impute to the 1000 genomes data, release 2010–06. SNPs with a quality score (Rsq) of <0.5 were discarded before analysis. The resulting genotypes were analyzed using PLINK with the same covariates for each dataset as the non-imputed analyses. The meta-analysis procedure was also identical to that described above.

### Ocular expression study of *LRP12* and *ZFPM2* in an animal model

#### Mouse laser capture microdissection

Retinas from adult mice were isolated, processed, and subjected to laser capture microdissection. cDNA purified from the respective cell layers was synthesized and analyzed by quantitative PCR as previously described [67].

#### Human ocular expression

Total RNA was extracted from dissected tissues (cornea, trabecular meshwork, retina, optic nerve) from normal human donor eyes as previously described [68]. Primer sequences were designed to specifically amplify the LRP12 and ZFPM2 genes. Amplification products were visualized by gel electrophoresis.

### DNA sequencing

Targeted resequencing using Sanger methods as described [69] was carried out on genes from the associated loci on chromosome 8 and chromosome 9. All exons of *LRP12* and *ZFPM2* were sequenced in 16 NPG patients, and all exons of *CDKN2BAS* were sequenced in 11 cases carrying a protective haplotype including rs2157719 (Table S6). Genomic DNA was sequenced using primers designed to amplify the coding exons as well as the adjacent splice sites for both genes. PCR products were directly sequenced on the ABI PRISM 3100 Genetic Analyzer (Applied Biosystems) with BigDye Terminators (Applied Biosystems) according to standard protocols. Methods and analysis for exome sequencing are described in Text S1.

### Association with exfoliation glaucoma

Exfoliation cases were collected from the Massachusetts Eye and Ear infirmary glaucoma clinic (cases) and the comprehensive eye service (controls in GLAUGEN or NEIGHBOR). All cases and controls were examined by a board certified ophthalmologist prior to study enrollment. The lead SNPs for each region associated with NPG were analyzed in 196 Caucasian patients with exfoliation syndrome, 104 of who also had glaucoma as defined as above. Single-SNP associations were analyzed using chi-square and logistic regression models adjusting for age.

### Web resources

PLINK (http://pngu.mgh.harvard.edu/~purcell/plink/); EIGENSOFT (http://genepath.med.harvard.edu/~reich/Software.htm); ENCODE (http://genome.ucsc.edu/ENCODE/); SCAN (http://www.scandb.org/newinterface/about.html); KEGG (http://www.genome.jp/kegg/); UCSC genome (http://genome.ucsc.edu/); ENSEMBL (http://useast.ensembl.org/index.html).

## Supporting Information

Figure S1 GLAUGEN QQ Plot. Quantile-quantile plots of P-values from using the logistic regression model that includes sex, age, study site (Nurses' Health Study, Health Professionals Follow-up Study and Genetic Etiology Primary Open-Angle Glaucoma), DNA source (blood or buccal), extraction method (DNAzol, Gentra or Qiagen), Eigen vectors 1, 2, and 6 as covariates.
(TIF)

Figure S2 GLAUGEN genome-wide associations with primary open-angle glaucoma (POAG). Results of the logistic regression case control analysis for the GLAUGEN dataset (976 cases and 1183 controls). The blue line represents a p-value of 1×10^−4^. SNP chromosome location is on the X axis and the p-value (−log10(p)) on the y axis. The logistic regression model includes sex, age, study site (Nurses' Health Study, Health Professionals Follow-up Study and Genetic Etiology Primary Open-Angle Glaucoma), DNA source (blood or buccal), extraction method (DNAzol, Gentra or Qiagen), Eigenvector (EV) 1, EV2, and EV6 as covariates.
(TIF)

Figure S3 NEIGHBOR QQ Plot. Quantile-quantile plots of p-values from using the logistic regression model that includes sex, age, study site (See Table S1), Eigenvector (EV)1, and EV2 as covariates.
(TIF)

Figure S4 NEIGHBOR genome-wide associations with primary open-angle glaucoma (POAG). Results of the logistic regression case control analysis for the NEIGHBOR dataset (2,517 cases and 2,428 controls). The red line identifies a p-value of 5×10^−8^. The logistic regression model includes sex, age, study site (See Table S1), Eigenvector (EV)1, and EV2 as covariates.
(TIF)

Figure S5 Genome-wide association results with primary open-angle glaucoma for the GLAUGEN-NEIGHBOR meta-analysis. The red line identifies a p value of 5×10^−8^. SNP chromosome location is on the X axis and the p-value (−log10(p)) on the y axis. Covariates include: (NEIGHBOR) age, gender, study site and eigenvectors 1 and 2; (GLAUGEN) age, gender, study site, DNA extraction method, DNA specimen type and eigenvectors 1, 2 and 6.
(TIF)

Figure S6 Genome-wide association results for High Pressure Glaucoma (HPG) in the GLAUGEN-NEIGHBOR meta-analysis. Results of the high-tension glaucoma (HTG) (IOP >22 mm Hg at or before diagnosis) meta-analysis (1669 cases and 3487 controls). The blue line identifies a p-value of 1×10^−4^. Covariates include: (NEIGHBOR) age, gender, study site and eigenvectors 1 and 2; (GLAUGEN) age, gender, study site, DNA extraction method, DNA specimen type and eigenvectors 1, 2 and 6.
(TIF)

Figure S7 9p21 genomic region associated with NPG. Depicted are predicted genes and splice variants for *CDKN2BAS*, *CDKN2B* and *CDKN2A* as seen in the UCSC Genome browser. Genotyped SNPs passing quality control measures that were not nominally significant in the NPG case-control analysis are colored black. SNPs that were nominally significant are colored blue (0.05<p<1×10^−4^), orange (1×10^−4^<p<5×10^−8^) and red (p<5×10^−8^).
(TIF)

Figure S8 8q22 genomic region associated with NPG. Depicted are predicted genes and splice variants for the 8q22 region as seen in the UCSC Genome browser. Genotyped SNPs passing quality control measures that were not nominally significant in association with normal pressure glaucoma in the case-control analysis are colored black. SNPs that were nominally significant are colored blue (0.05<p<1×10−4), orange (1×10−4<p<5×10−8) and red (p<5×10−8).
(TIF)

Figure S9 DNaseI signals for various cell types in a portion of the chromosome 9p21 region associated with NPG. The location of the lead SNP in this region rs2157719 is highlighted. Of the 120 cell types considered the lead SNP overlaps DNaseI sites in six of them. Two signal tracks for each cell line are presented. Vertebrate sequence homology, a chromatin state segmentation where orange indicates candidate strong enhancers, and GERP (Genomic Evolutionary Rate Profiling) scores are shown below the SNP locations. Abbreviations: HBMEC, brain microvascular endothelial cells; HEEpiC, esophageal epithelial cells; HMEC, mammary epithelial cells; LNCaP, prostate cancer cells; NHEK, epidermal keratinocytes; SAEC, small airway epithelial cells; WI-38 embryonic lung fibroblast cells; HPDE6, pancreatic duct cells.
(TIF)

Figure S10 DNaseI signals for various cell types in the chromosome 8q22 region associated with NPG. Of the 120 cell types tested, one SNP, rs284492, in strong LD (r^2^>0.8) with the lead SNP, rs284489, overlapped a DNaseI site. The figure shows the 10 cell types with DNaseI sites overlapping or proximal to rs284492. Two signals for each cell line are presented. Vertebrate sequence homology, the locations of the DNaseI hypersensitivity sites and GERP (Genomic Evolutionary Rate Profiling) scores are shown below the SNP locations. Abbreviations: HAc, astrocytes-cerebellar; HA-h, astrocytes-hippocampal; HA-sp, astrocytes spinal cord; HBMEC, brain microvascular endothelial cells; HCPEpiC, choroid plexus epithelial cells; HIPEpiC, iris pigment epithelial cells; HNPCEpiC, non-pigment ciliary epithelial cells; NH-A astrocytes; NHLF, lung fibroblasts; WERI-RB-1 retinoblastoma.
(TIF)

Figure S11 Ocular expression studies for LRP12 and ZFPM2. Ocular expression studies for *LRP12* and *ZFPM2*. Panel A, Ocular expression in tissues dissected from two human cadaver eyes. Panel B. Quantitative PCR results from laser microdissection in mouse retina. The y-axis is the abundance of the mRNA relative to Gapdh for each cell layer. Each bar represents the average of 3 (GCL, INL) and 4 (ONL) independent adult mouse samples. Abbreviations: Cor, cornea; Tm, trabecular meshwork; On, optic nerve; Ret, retina; GCL, ganglion cell layer; INL, inner nuclear layer; ONL, outer nuclear layer.
(TIF)

Table S1 Sites contributing cases and controls to the NEIGHBOR GWAS. Cases and controls were recruited from ophthalmology clinics (West Virginia University, University of Pittsburgh, Johns Hopkins Medical School, Stanford University, University of California, San Diego, University of Miami, Duke University, University of Michigan, the Marshfield Clinic) and were examined by ophthalmologists, using case and control definitions that were harmonized with the GLAUGEN study. Cases and controls were also drawn from two clinical trial populations: Advanced Glaucoma Intervention Study (AGIS, NEI U10EY006827, D. Gaasterland PI) and the Collaborative Initial Glaucoma Treatment Study (CIGTS, NEI U10 EY009149, P. Lichter PI). An additional set of 1496 controls, individuals who had been examined at the Duke Eye Center, were selected from among 7500 subjects who had undergone cardiac catheterization at Duke Medical Center and who are part of the CATHGEN biorepository at Duke with blood samples collected at the time of catheterization.
(DOCX)

Table S2 Association results for genome-wide significant SNPs (p<5×10−8) associated with POAG for the NEIGHBOR GWAS. Chr = chromosome, BP = genomic position in basepair, MAF = minor allele frequency, OR = odds ratio, L95/U95 = 95% confidence interval lower and upper limits.
(DOCX)

Table S3 Association results for genome-wide significant SNPs (p<5×10−8) associated with POAG for the GLAUGEN NEIGHBOR meta-analysis. Chr = chromosome, BP = genomic position in basepair, MAF = minor allele frequency, OR = odds ratio, L95/U95 = 95% confidence interval lower and upper limits.
(DOCX)

Table S4 Association results from the NEIGHBOR-GLAUGEN meta-analysis for SNPs located in genomic regions previously associated with optic nerve parameters. Alleles 1 and 2 are listed alphabetically. P values and OR (odds ratio) are listed for POAG (primary open angle glaucoma) overall as well as for HPG (high pressure glaucoma) and NPG (normal pressure glaucoma). The effect allele is the minor allele. The direction column refers to the direction of effect in the individual GLAUGEN and NEIGHBOR datasets. The GLAUGEN direction is listed first and the NEIGHBOR direction is listed second. A minus sign signifies an OR less than one while a plus sign indicates an OR greater than one.
(XLSX)

Table S5 SNPs associated with NPG (p<5×10−8) in the 9p21 and 8q22 regions after imputation. Alleles 1 and 2 are listed alphabetically. P values and OR (odds ratio) are listed for and NPG (normal pressure glaucoma) after imputation for the GLAUGEN-NEIGHBOR meta-analysis. The effect allele is the minor allele. The direction column refers to the direction of effect in the individual GLAUGEN and NEIGHBOR datasets. The GLAUGEN direction is listed first and the NEIGHBOR direction is listed second. A minus sign signifies an OR less than one while a plus sign indicates an OR greater than one.
(DOCX)

Table S6 CDKN2BAS haplotypes for the NEIGHBOR and GLAUGEN NPG datasets. Haplotype analysis of SNPs that were nominally significant in the CDKN2BAS regions in the NEIGHBOR and GLAUGEN NPG datasets. The haplotype with the most significant association is indicated in bold text. Abbreviations: Bp (base pair); Freq (frequency); NPG (normal pressure glaucoma); OR (odds ratio).
(DOCX)

Table S7 Haplotypes for 8q22 associated region for the NEIGHBOR and GLAUGEN NPG datasets. Haplotype analysis of SNPs that were nominally significant in the 8q22 region in the NEIGHBOR and GLAUGEN NPG datasets. Abbreviations: Bp, base pair; Freq, frequency; NPG, normal pressure glaucoma; OR, odds ratio.
(DOCX)

Table S8 DNA sequence variants in LRP12 and ZFPM2 in NPG and POAG patients and controls. Abbreviations: Ref, reference allele; Obs, observed alleles; POAG, primary open angle glaucoma; NPG, normal pressure glaucoma, AA, amino acid; UTR-5, 5′ untranslated region; UTR-3, 3′ untranslated region; SYNON, synonymous.
(DOCX)

Text S1 Supplemental Methods for exome sequencing and analysis.
(DOCX)
