# Supplementary figures and images for: Common Variants at 9p21 and 8q22 Are Associated with Increased Susceptibility to Optic Nerve Degeneration in Glaucoma
Source: PLoS Genet. 2012 Apr 26;8(4):e1002654. doi: 10.1371/journal.pgen.1002654 (PMC3343074; doi:10.1371/journal.pgen.1002654)

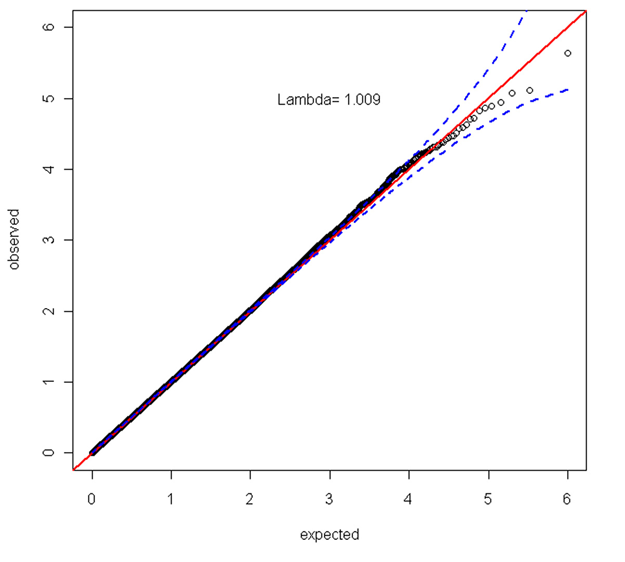

Supplement: Figure S1 — GLAUGEN QQ Plot. Quantile-quantile plots of P-values from using the logistic regression model that includes sex, age, study site (Nurses' Health Study, Health Professionals Follow-up Study and Genetic Etiology Primary Open-Angle Glaucoma), DNA source (blood or buccal), extraction method (DNAzol, Gentra or Qiagen), Eigen vectors 1, 2, and 6 as covariates. (TIF) [file pgen.1002654.s001.tif]

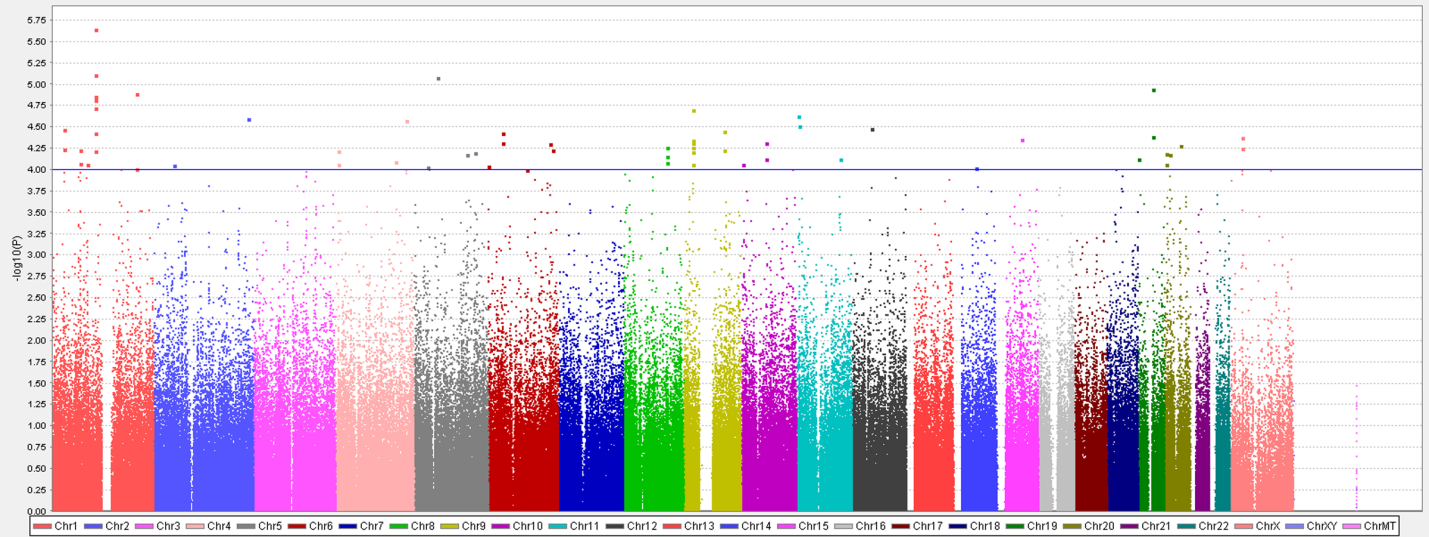

Supplement: Figure S2 — GLAUGEN genome-wide associations with primary open-angle glaucoma (POAG). Results of the logistic regression case control analysis for the GLAUGEN dataset (976 cases and 1183 controls). The blue line represents a p-value of 1×10^−4^. SNP chromosome location is on the X axis and the p-value (−log10(p)) on the y axis. The logistic regression model includes sex, age, study site (Nurses' Health Study, Health Professionals Follow-up Study and Genetic Etiology Primary Open-Angle Glaucoma), DNA source (blood or buccal), extraction method (DNAzol, Gentra or Qiagen), Eigenvector (EV) 1, EV2, and EV6 as covariates. (TIF) [file pgen.1002654.s002.tif]

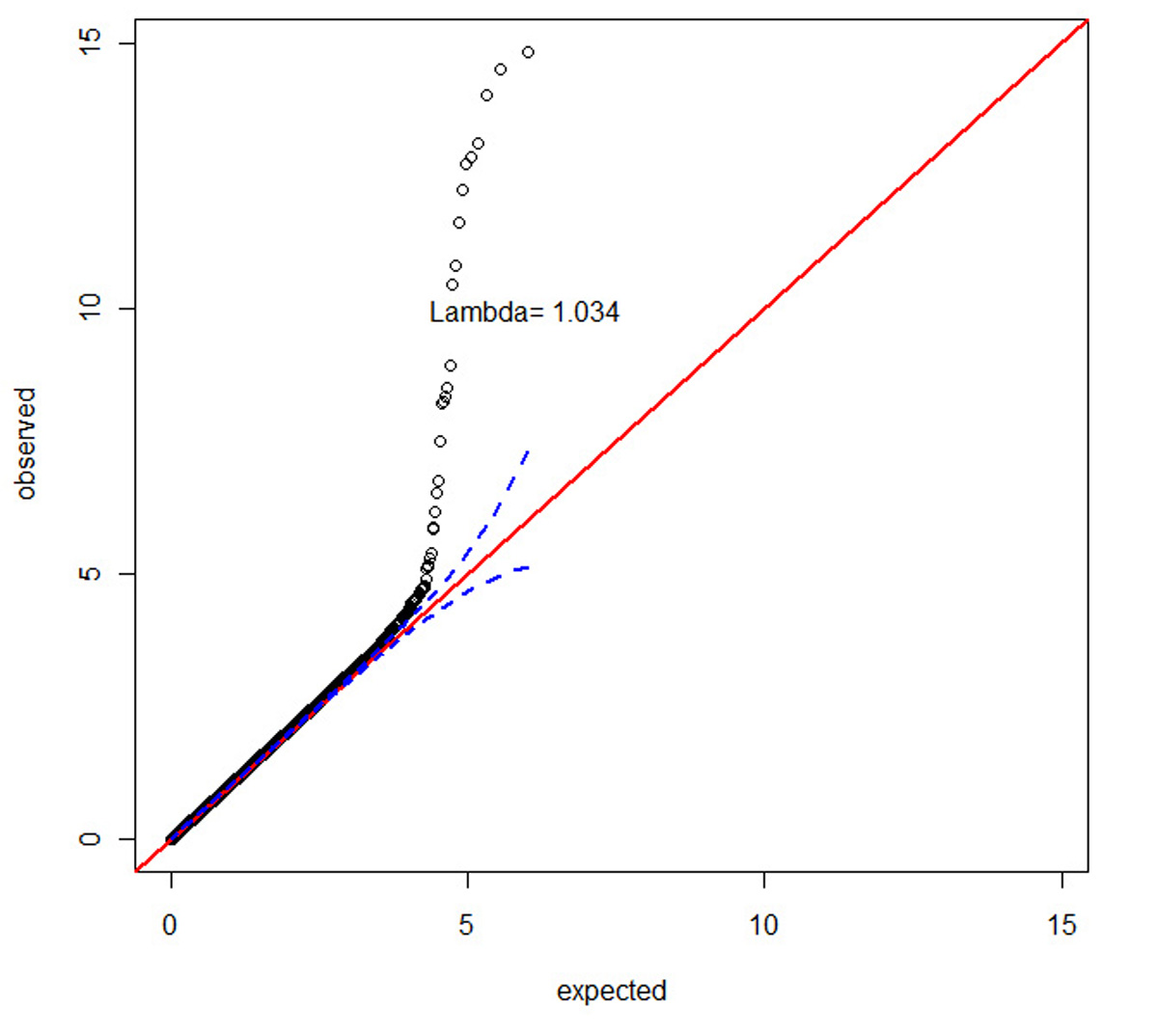

Supplement: Figure S3 — NEIGHBOR QQ Plot. Quantile-quantile plots of p-values from using the logistic regression model that includes sex, age, study site (See Table S1), Eigenvector (EV)1, and EV2 as covariates. (TIF) [file pgen.1002654.s003.tif]

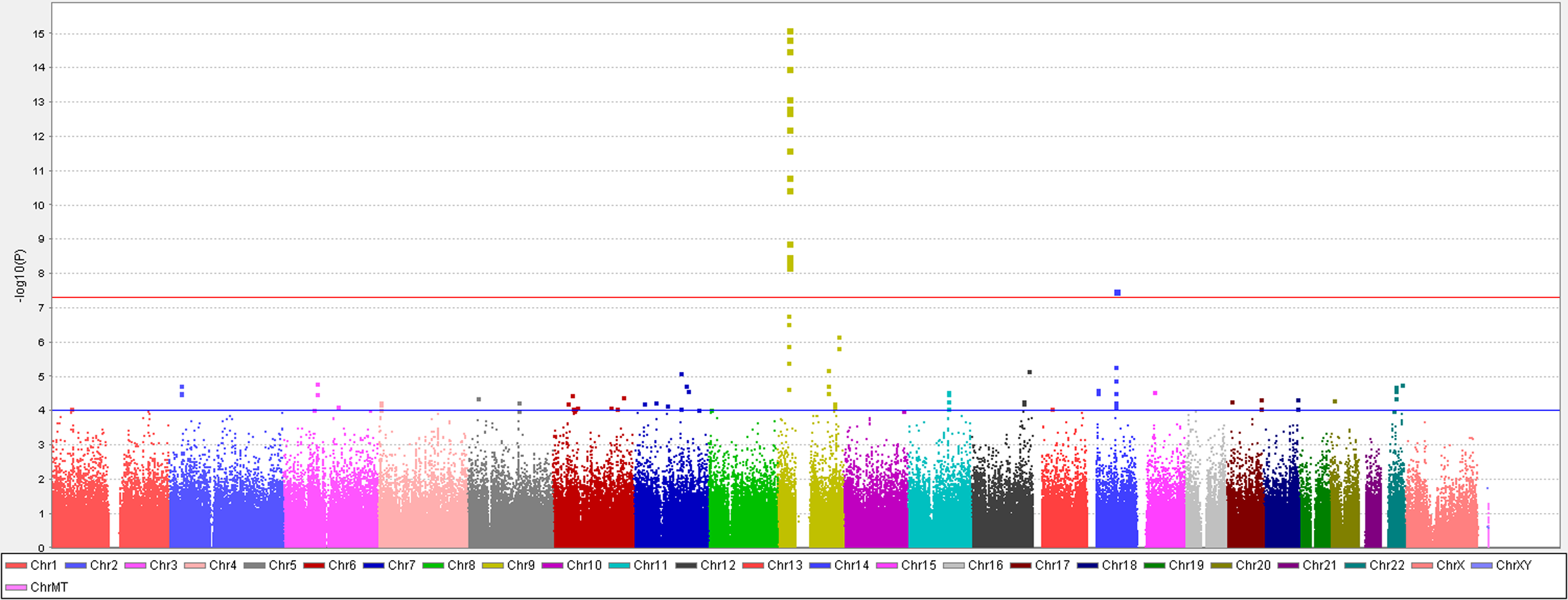

Supplement: Figure S4 — NEIGHBOR genome-wide associations with primary open-angle glaucoma (POAG). Results of the logistic regression case control analysis for the NEIGHBOR dataset (2,517 cases and 2,428 controls). The red line identifies a p-value of 5×10^−8^. The logistic regression model includes sex, age, study site (See Table S1), Eigenvector (EV)1, and EV2 as covariates. (TIF) [file pgen.1002654.s004.tif]

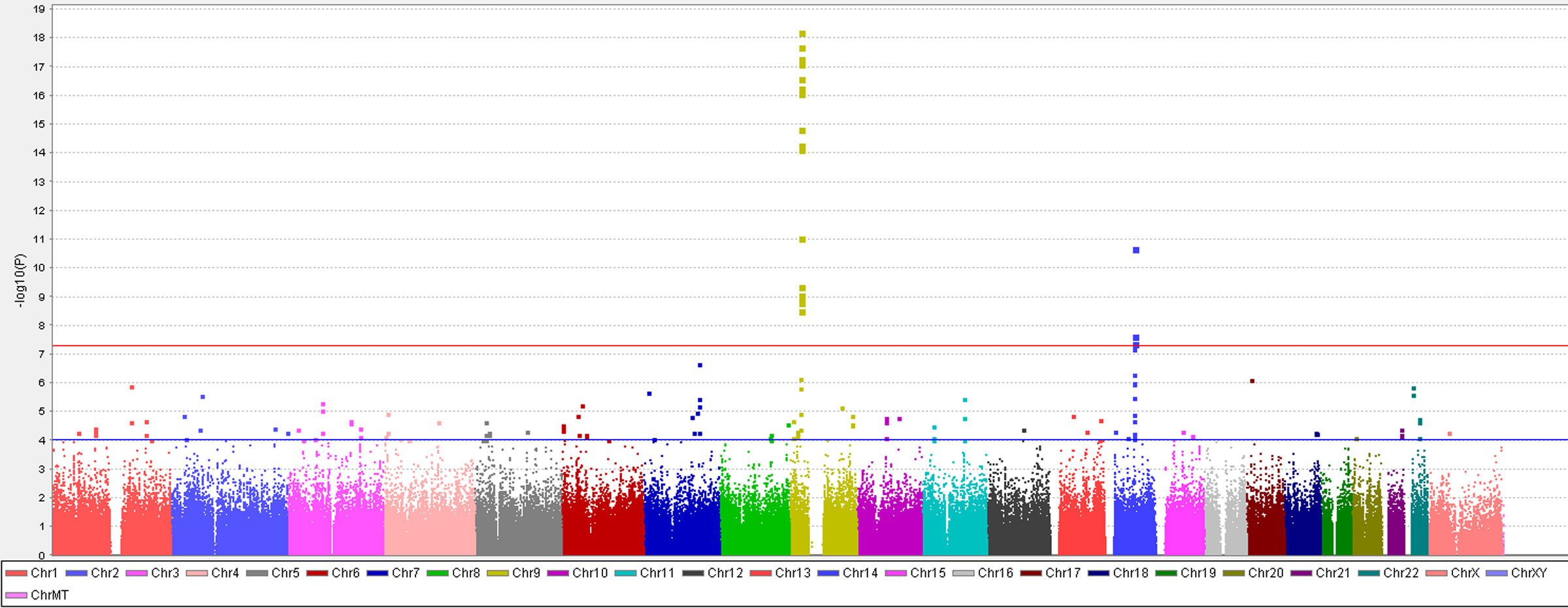

Supplement: Figure S5 — Genome-wide association results with primary open-angle glaucoma for the GLAUGEN-NEIGHBOR meta-analysis. The red line identifies a p value of 5×10^−8^. SNP chromosome location is on the X axis and the p-value (−log10(p)) on the y axis. Covariates include: (NEIGHBOR) age, gender, study site and eigenvectors 1 and 2; (GLAUGEN) age, gender, study site, DNA extraction method, DNA specimen type and eigenvectors 1, 2 and 6. (TIF) [file pgen.1002654.s005.tif]

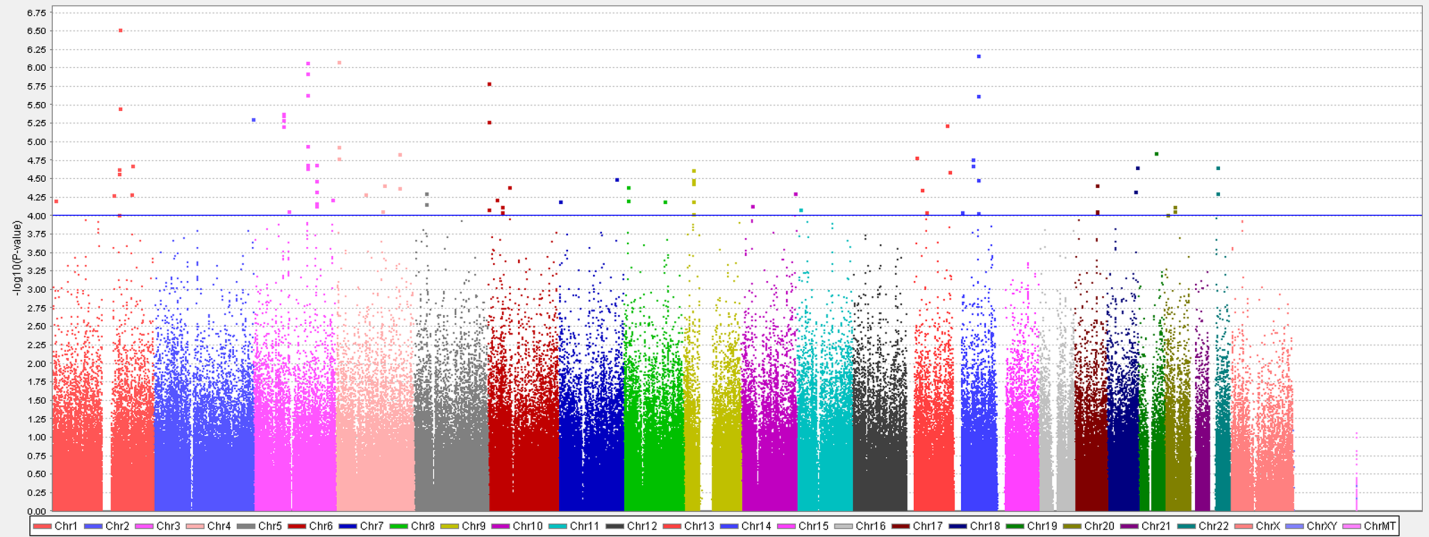

Supplement: Figure S6 — Genome-wide association results for High Pressure Glaucoma (HPG) in the GLAUGEN-NEIGHBOR meta-analysis. Results of the high-tension glaucoma (HTG) (IOP >22 mm Hg at or before diagnosis) meta-analysis (1669 cases and 3487 controls). The blue line identifies a p-value of 1×10^−4^. Covariates include: (NEIGHBOR) age, gender, study site and eigenvectors 1 and 2; (GLAUGEN) age, gender, study site, DNA extraction method, DNA specimen type and eigenvectors 1, 2 and 6. (TIF) [file pgen.1002654.s006.tif]

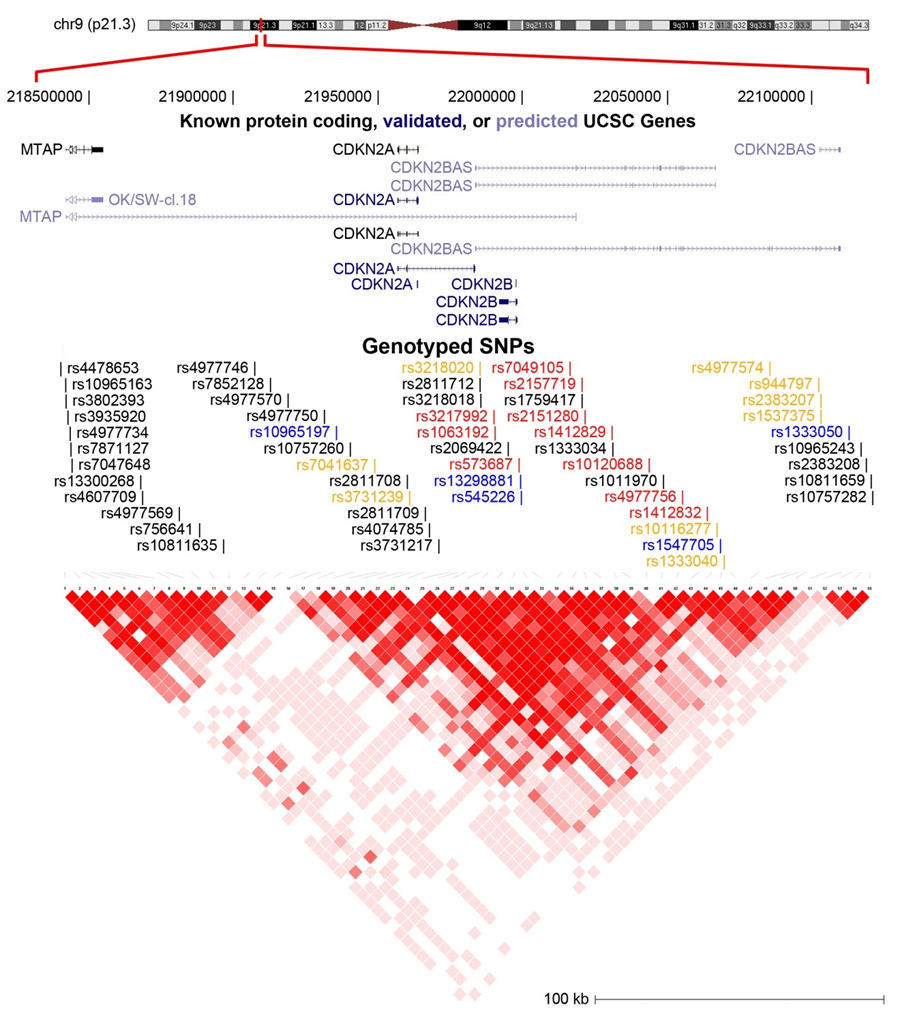

Supplement: Figure S7 — 9p21 genomic region associated with NPG. Depicted are predicted genes and splice variants for CDKN2BAS, CDKN2B and CDKN2A as seen in the UCSC Genome browser. Genotyped SNPs passing quality control measures that were not nominally significant in the NPG case-control analysis are colored black. SNPs that were nominally significant are colored blue (0.05<p<1×10^−4^), orange (1×10^−4^<p<5×10^−8^) and red (p<5×10^−8^). (TIF) [file pgen.1002654.s007.tif]

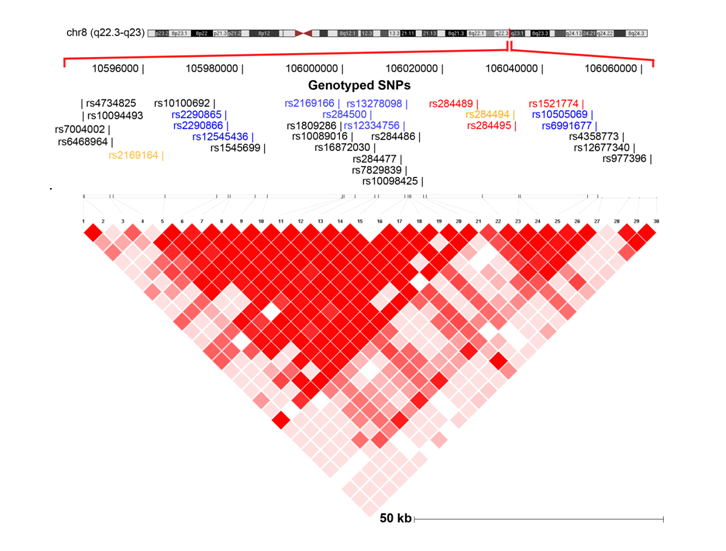

Supplement: Figure S8 — 8q22 genomic region associated with NPG. Depicted are predicted genes and splice variants for the 8q22 region as seen in the UCSC Genome browser. Genotyped SNPs passing quality control measures that were not nominally significant in association with normal pressure glaucoma in the case-control analysis are colored black. SNPs that were nominally significant are colored blue (0.05<p<1×10^−4^), orange (1×10^−4^<p<5×10^−8^) and red (p<5×10^−8^). (TIF) [file pgen.1002654.s008.tif]

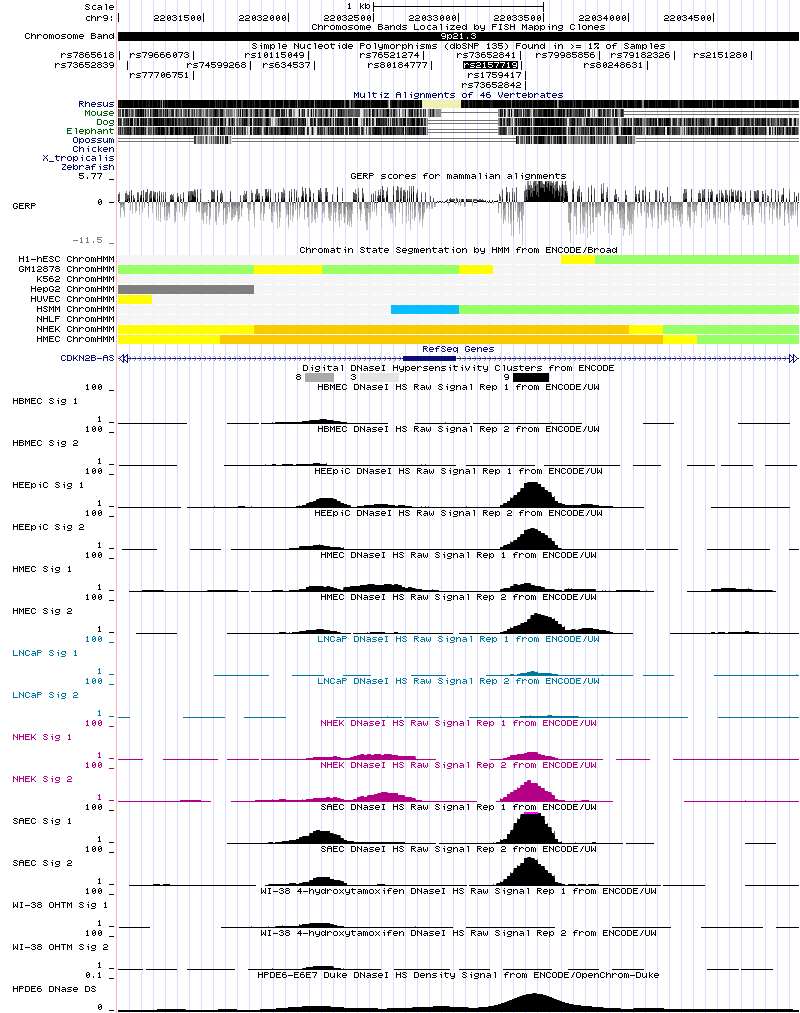

Supplement: Figure S9 — DNaseI signals for various cell types in a portion of the chromosome 9p21 region associated with NPG. The location of the lead SNP in this region rs2157719 is highlighted. Of the 120 cell types considered the lead SNP overlaps DNaseI sites in six of them. Two signal tracks for each cell line are presented. Vertebrate sequence homology, a chromatin state segmentation where orange indicates candidate strong enhancers, and GERP (Genomic Evolutionary Rate Profiling) scores are shown below the SNP locations. Abbreviations: HBMEC, brain microvascular endothelial cells; HEEpiC, esophageal epithelial cells; HMEC, mammary epithelial cells; LNCaP, prostate cancer cells; NHEK, epidermal keratinocytes; SAEC, small airway epithelial cells; WI-38 embryonic lung fibroblast cells; HPDE6, pancreatic duct cells. (TIF) [file pgen.1002654.s009.tif]

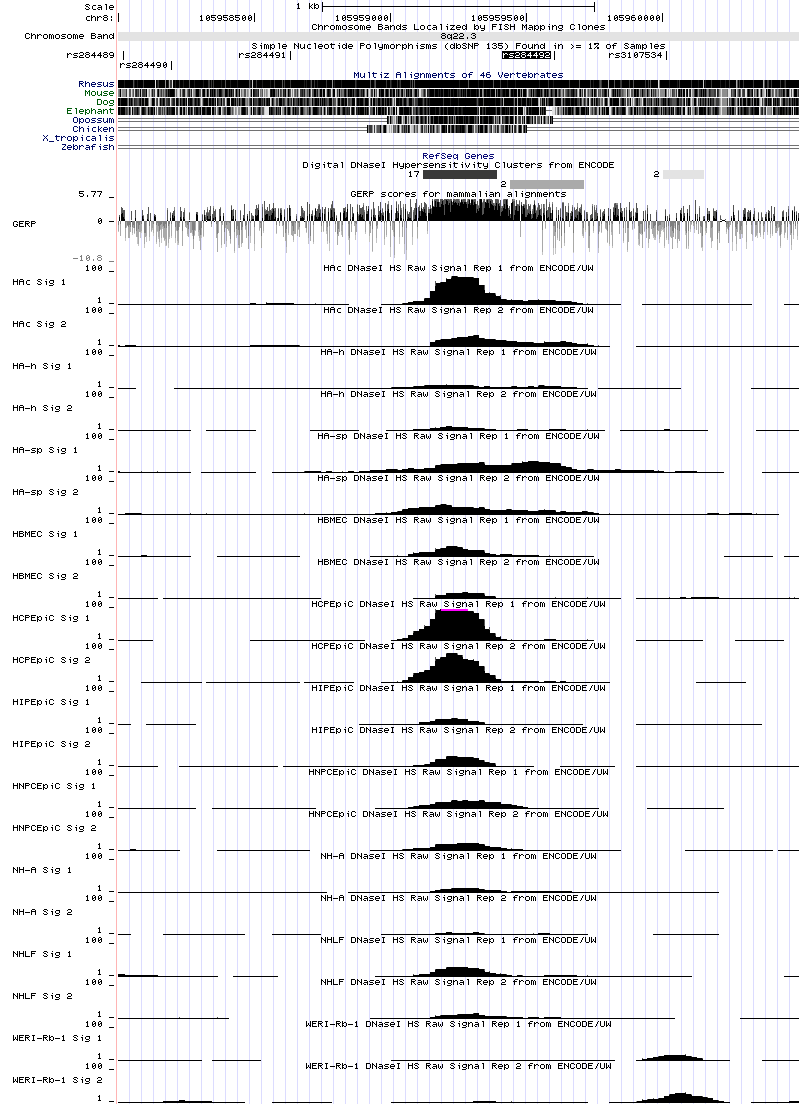

Supplement: Figure S10 — DNaseI signals for various cell types in the chromosome 8q22 region associated with NPG. Of the 120 cell types tested, one SNP, rs284492, in strong LD (r2>0.8) with the lead SNP, rs284489, overlapped a DNaseI site. The figure shows the 10 cell types with DNaseI sites overlapping or proximal to rs284492. Two signals for each cell line are presented. Vertebrate sequence homology, the locations of the DNaseI hypersensitivity sites and GERP (Genomic Evolutionary Rate Profiling) scores are shown below the SNP locations. Abbreviations: HAc, astrocytes-cerebellar; HA-h, astrocytes-hippocampal; HA-sp, astrocytes spinal cord; HBMEC, brain microvascular endothelial cells; HCPEpiC, choroid plexus epithelial cells; HIPEpiC, iris pigment epithelial cells; HNPCEpiC, non-pigment ciliary epithelial cells; NH-A astrocytes; NHLF, lung fibroblasts; WERI-RB-1 retinoblastoma. (TIF) [file pgen.1002654.s010.tif]

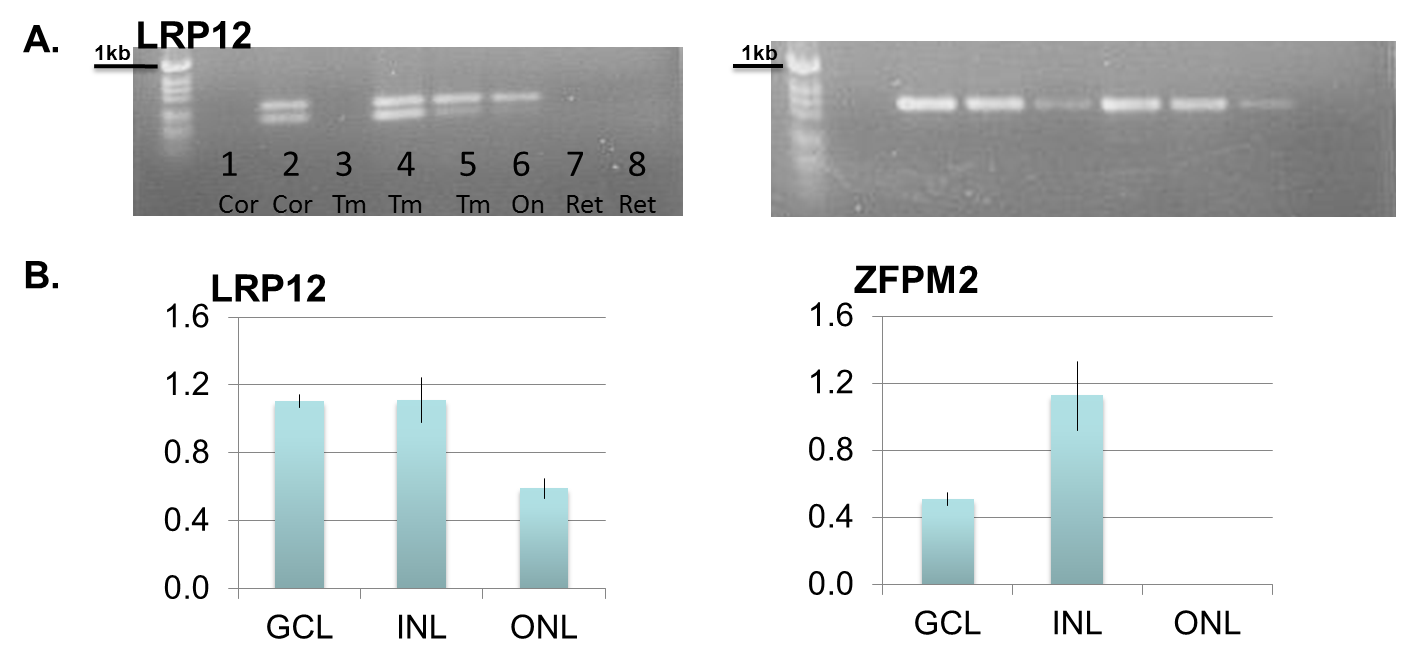

Supplement: Figure S11 — Ocular expression studies for LRP12 and ZFPM2. Ocular expression studies for LRP12 and ZFPM2. Panel A, Ocular expression in tissues dissected from two human cadaver eyes. Panel B. Quantitative PCR results from laser microdissection in mouse retina. The y-axis is the abundance of the mRNA relative to Gapdh for each cell layer. Each bar represents the average of 3 (GCL, INL) and 4 (ONL) independent adult mouse samples. Abbreviations: Cor, cornea; Tm, trabecular meshwork; On, optic nerve; Ret, retina; GCL, ganglion cell layer; INL, inner nuclear layer; ONL, outer nuclear layer. (TIF) [file pgen.1002654.s011.tif]
