# Supplementary material for: Common Variants at 9p21 and 8q22 Are Associated with Increased Susceptibility to Optic Nerve Degeneration in Glaucoma
Source: PLoS Genet. 2012 Apr 26;8(4):e1002654. doi: 10.1371/journal.pgen.1002654 (PMC3343074; doi:10.1371/journal.pgen.1002654)
Supplement: Table S1 — Sites contributing cases and controls to the NEIGHBOR GWAS. Cases and controls were recruited from ophthalmology clinics (West Virginia University, University of Pittsburgh, Johns Hopkins Medical School, Stanford University, University of California, San Diego, University of Miami, Duke University, University of Michigan, the Marshfield Clinic) and were examined by ophthalmologists, using case and control definitions that were harmonized with the GLAUGEN study. Cases and controls were also drawn from two clinical trial populations: Advanced Glaucoma Intervention Study (AGIS, NEI U10EY006827, D. Gaasterland PI) and the Collaborative Initial Glaucoma Treatment Study (CIGTS, NEI U10 EY009149, P. Lichter PI). An additional set of 1496 controls, individuals who had been examined at the Duke Eye Center, were selected from among 7500 subjects who had undergone cardiac catheterization at Duke Medical Center and who are part of the CATHGEN biorepository at Duke with blood samples collected at the time of catheterization. (DOCX) [file pgen.1002654.s012.docx]

**Table S1. Sites contributing cases and controls to the NEIGHBOR GWAS**

Site Cases Controls Total

| University of West Virginia | 173 | 100 | 273 |
| --- | --- | --- | --- |
| University of Pittsburgh | 124 | 64 | 188 |
| Johns Hopkins Medical School | 48 | 96 | 144 |
| Stanford University | 37 | 0 | 37 |
| University of California, San Diego | 839 | 640 | 1479 |
| University of Miami | 244 | 229 | 473 |
| Duke University Medical School, Ophthalmology clinics | 414 | 539 | 953 |
| Duke University CATHGEN Study | 0 | 548 | 548 |
| University of Michigan | 292 | 66 | 358 |
| Michigan: CIGITS study | 221 | 0 | 221 |
| Michigan: AGIS study | 45 | 0 | 45 |
| Marshfield Clinic | 80 | 146 | 226 |
| Totals | 2517 | 2428 | 4945 |

Cases and controls were recruited from ophthalmology clinics (West Virginia University, University of Pittsburgh, Johns Hopkins Medical School, Stanford University, University of California, San Diego, University of Miami, Duke University, University of Michigan, the Marshfield Clinic) and were examined by ophthalmologists, using case and control definitions that were harmonized with the GLAUGEN study. Cases and controls were also drawn from two clinical trial populations: Advanced Glaucoma Intervention Study (AGIS, NEI U10EY006827, D. Gaasterland PI) and the Collaborative Initial Glaucoma Treatment Study (CIGTS, NEI U10 EY009149, P. Lichter PI). An additional set of 1496 controls, individuals who had been examined at the Duke Eye Center, were selected from among 7500 subjects who had undergone cardiac catheterization at Duke Medical Center and who are part of the CATHGEN biorepository at Duke with blood samples collected at the time of catheterization.
