# Supplementary material for: Common Variants at 9p21 and 8q22 Are Associated with Increased Susceptibility to Optic Nerve Degeneration in Glaucoma
Source: PLoS Genet. 2012 Apr 26;8(4):e1002654. doi: 10.1371/journal.pgen.1002654 (PMC3343074; doi:10.1371/journal.pgen.1002654)
Supplement: Table S2 — Association results for genome-wide significant SNPs (p<5×10^−8^) associated with POAG for the NEIGHBOR GWAS. Chr = chromosome, BP = genomic position in basepair, MAF = minor allele frequency, OR = odds ratio, L95/U95 = 95% confidence interval lower and upper limits. (DOCX) [file pgen.1002654.s013.docx]

**Table S2. Association results for genome-wide significant SNPs (p<5x10^-8^) associated with POAG for the NEIGHBOR GWAS**

| SNP | CHR | Minor Allele | MAF Cases | MAF Controls | OR | L95 | U95 | P | Gene |
| --- | --- | --- | --- | --- | --- | --- | --- | --- | --- |
| rs4977756 | 9 | G | 0.31 | 0.40 | 0.66 | 0.59 | 0.73 | 7.34E-16 | CDKN2BAS |
| rs2157719 | 9 | G | 0.33 | 0.42 | 0.67 | 0.60 | 0.74 | 1.39E-15 | CDKN2BAS |
| rs1412829 | 9 | G | 0.32 | 0.42 | 0.67 | 0.60 | 0.74 | 2.95E-15 | CDKN2BAS |
| rs1063192 | 9 | G | 0.34 | 0.43 | 0.67 | 0.61 | 0.75 | 9.67E-15 | CDKN2B, CDKN2BAS, MTAP |
| rs7049105 | 9 | A | 0.43 | 0.52 | 0.69 | 0.63 | 0.76 | 7.38E-14 | CDKN2BAS, MTAP |
| rs10120688 | 9 | G | 0.42 | 0.50 | 0.69 | 0.63 | 0.76 | 1.42E-13 | CDKN2BAS |
| rs2151280 | 9 | G | 0.44 | 0.52 | 0.70 | 0.63 | 0.77 | 1.87E-13 | CDKN2BAS |
| rs573687 | 9 | A | 0.29 | 0.38 | 0.68 | 0.62 | 0.76 | 5.64E-13 | CDKN2BAS, MTAP |
| rs1412832 | 9 | G | 0.24 | 0.31 | 0.68 | 0.61 | 0.76 | 2.36E-12 | CDKN2BAS |
| rs3217992 | 9 | A | 0.46 | 0.39 | 1.40 | 1.27 | 1.54 | 1.48E-11 | CDKN2B, CDKN2BAS, MTAP |
| rs10116277 | 9 | C | 0.44 | 0.51 | 0.72 | 0.66 | 0.80 | 3.45E-11 | CDKN2BAS |
| rs2383207 | 9 | A | 0.41 | 0.48 | 0.74 | 0.67 | 0.82 | 1.20E-09 | CDKN2BAS |
| rs3218020 | 9 | A | 0.43 | 0.36 | 1.35 | 1.22 | 1.49 | 3.06E-09 | CDKN2BAS, MTAP |
| rs4977574 | 9 | A | 0.44 | 0.51 | 0.75 | 0.68 | 0.83 | 4.33E-09 | CDKN2BAS |
| rs1537375 | 9 | A | 0.42 | 0.49 | 0.75 | 0.68 | 0.83 | 5.75E-09 | CDKN2BAS |
| rs944797 | 9 | A | 0.42 | 0.48 | 0.75 | 0.68 | 0.83 | 5.94E-09 | CDKN2BAS |
| rs10483727 | 14 | A | 0.46 | 0.40 | 1.32 | 1.20 | 1.46 | 3.10E-08 | Intergenic - SIX1/SIX6 |

**Chr=** chromosome, **BP=** genomic position in basepair, **MAF=** minor allele frequency, **OR=** odds ratio, **L95/U95=** 95% confidence interval lower and upper limits.
