# Supplementary material for: Common Variants at 9p21 and 8q22 Are Associated with Increased Susceptibility to Optic Nerve Degeneration in Glaucoma
Source: PLoS Genet. 2012 Apr 26;8(4):e1002654. doi: 10.1371/journal.pgen.1002654 (PMC3343074; doi:10.1371/journal.pgen.1002654)
Supplement: Table S3 — Association results for genome-wide significant SNPs (p<5×10^−8^) associated with POAG for the GLAUGEN NEIGHBOR meta-analysis. Chr = chromosome, BP = genomic position in basepair, MAF = minor allele frequency, OR = odds ratio, L95/U95 = 95% confidence interval lower and upper limits. (DOCX) [file pgen.1002654.s014.docx]

**Table S3. Association results for genome-wide significant SNPs (p<5x10^-8^) associated with POAG for the GLAUGEN NEIGHBOR meta-analysis**

| SNP | CHR | BP | Minor Allele | OR | L95 | U95 | P | Gene |
| --- | --- | --- | --- | --- | --- | --- | --- | --- |
| rs2157719 | 9 | 22023366 | G | 0.69 | 0.63 | 0.75 | 1.86E-18 | CDKN2BAS |
| rs4977756 | 9 | 22058652 | G | 0.69 | 0.63 | 0.75 | 6.21E-18 | CDKN2BAS |
| rs1412829 | 9 | 22033926 | G | 0.69 | 0.64 | 0.76 | 1.46E-17 | CDKN2BAS |
| rs1063192 | 9 | 21993367 | G | 0.70 | 0.64 | 0.76 | 2.30E-17 | CDKN2B, CDKN2BAS, MTAP |
| rs7049105 | 9 | 22018801 | A | 0.71 | 0.65 | 0.77 | 6.94E-17 | CDKN2BAS, MTAP |
| rs10120688 | 9 | 22046499 | G | 0.71 | 0.65 | 0.77 | 1.41E-16 | CDKN2BAS |
| rs2151280 | 9 | 22024719 | G | 0.71 | 0.66 | 0.78 | 2.15E-16 | CDKN2BAS |
| rs3217992 | 9 | 21993223 | A | 1.39 | 1.28 | 1.51 | 3.34E-15 | CDKN2B, CDKN2BAS, MTAP |
| rs573687 | 9 | 22001642 | A | 0.71 | 0.65 | 0.78 | 1.28E-14 | CDKN2BAS, MTAP |
| rs1412832 | 9 | 22067543 | G | 0.70 | 0.64 | 0.77 | 1.75E-14 | CDKN2BAS |
| rs10116277 | 9 | 22071397 | C | 0.76 | 0.70 | 0.83 | 1.78E-11 | CDKN2BAS |
| rs10483727 | 14 | 60142628 | A | 1.32 | 1.21 | 1.43 | 3.87E-11 | Intergenic - SIX1/SIX6 |
| rs4977574 | 9 | 22088574 | A | 0.78 | 0.72 | 0.84 | 7.20E-10 | CDKN2BAS |
| rs2383207 | 9 | 22105959 | A | 0.78 | 0.72 | 0.85 | 1.50E-09 | CDKN2BAS |
| rs1537375 | 9 | 22106071 | A | 0.78 | 0.72 | 0.85 | 2.16E-09 | CDKN2BAS |
| rs944797 | 9 | 22105286 | A | 0.78 | 0.72 | 0.85 | 2.62E-09 | CDKN2BAS |
| rs1333040 | 9 | 22073404 | G | 0.78 | 0.72 | 0.85 | 4.88E-09 | CDKN2BAS |
| rs3218020 | 9 | 21987872 | A | 1.35 | 1.22 | 1.49 | 5.49E-09 | CDKN2BAS, MTAP |
| rs4901977 | 14 | 59858929 | A | 1.27 | 1.17 | 1.39 | 3.37E-08 | Intergenic - SIX1/SIX6 |

**Chr=** chromosome, **BP=** genomic position in basepair, **MAF=** minor allele frequency, **OR=** odds ratio, **L95/U95=** 95% confidence interval lower and upper limits.
