# Supplementary material for: Common Variants at 9p21 and 8q22 Are Associated with Increased Susceptibility to Optic Nerve Degeneration in Glaucoma
Source: PLoS Genet. 2012 Apr 26;8(4):e1002654. doi: 10.1371/journal.pgen.1002654 (PMC3343074; doi:10.1371/journal.pgen.1002654)
Supplement: Table S5 — SNPs associated with NPG (p<5×10^−8^) in the 9p21 and 8q22 regions after imputation. Alleles 1 and 2 are listed alphabetically. P values and OR (odds ratio) are listed for and NPG (normal pressure glaucoma) after imputation for the GLAUGEN-NEIGHBOR meta-analysis. The effect allele is the minor allele. The direction column refers to the direction of effect in the individual GLAUGEN and NEIGHBOR datasets. The GLAUGEN direction is listed first and the NEIGHBOR direction is listed second. A minus sign signifies an OR less than one while a plus sign indicates an OR greater than one. (DOCX) [file pgen.1002654.s016.docx]

**Table S5. SNPs associated with NPG (p < 5x10^-8^) in the 9p21 and 8q22 regions after imputation.**

**8q22**

| SNP | Allele1 | Allele2 | Effect | OR | StdErr | P-value | Direction | Rsq-Neighbor | Rsq-Glaugen |
| --- | --- | --- | --- | --- | --- | --- | --- | --- | --- |
| rs284488 | a | g | 0.4818 | 1.62 | 0.0781 | 6.92E-10 | ++ | 0.9909 | 0.9862 |
| rs284490 | a | c | 0.4818 | 1.62 | 0.0781 | 6.92E-10 | ++ | 0.9981 | 0.9998 |
| rs284487 | t | c | -0.4818 | 0.62 | 0.0781 | 6.92E-10 | -- | 0.9889 | 0.9843 |
| rs1851292 | t | c | 0.4818 | 1.62 | 0.0781 | 6.92E-10 | ++ | 0.9852 | 0.9823 |
| rs284489 | a | g | 0.4818 | 1.62 | 0.0781 | 6.92E-10 | ++ | 0.9983 | 0.9998 |
| rs284491 | t | c | -0.4794 | 0.62 | 0.0784 | 9.71E-10 | -- | 0.9944 | 0.9993 |
| rs1521774 | a | g | 0.4765 | 1.61 | 0.0784 | 1.19E-09 | ++ | 1 | 1 |
| rs1521772 | a | g | 0.4737 | 1.61 | 0.0782 | 1.42E-09 | ++ | 1 | 1 |
| rs284495 | t | c | 0.4737 | 1.61 | 0.0782 | 1.42E-09 | ++ | 1 | 1 |
| rs284492 | t | c | -0.4737 | 0.62 | 0.0782 | 1.42E-09 | -- | 0.9972 | 0.9998 |
| rs284496 | a | c | 0.4722 | 1.60 | 0.0784 | 1.67E-09 | ++ | 0.9907 | 0.9878 |
| rs1521771 | a | t | 0.4682 | 1.60 | 0.0783 | 2.26E-09 | ++ | 0.9833 | 0.9799 |
| rs10106029 | a | g | -0.4568 | 0.63 | 0.0785 | 6.03E-09 | -- | 0.8712 | 0.8576 |

**9p21**

| SNP | | Allele1 | Allele2 | BETA | OR | StdErr | P-value | Direction | Rsq-Glaugen | Rsq-Neighbor |
| --- | --- | --- | --- | --- | --- | --- | --- | --- | --- | --- |
| rs7865618 | | a | g | 0.5632 | 1.76 | 0.0769 | 2.48E-13 | ++ | 0.9982 | 0.9926 |
| rs2157719 | | t | c | 0.5536 | 1.74 | 0.0768 | 5.84E-13 | ++ | 0.9999 | 0.9979 |
| rs634537 | | t | g | 0.551 | 1.73 | 0.0776 | 1.24E-12 | ++ | 0.9993 | 0.9918 |
| rs564398 | | t | c | 0.551 | 1.73 | 0.0776 | 1.24E-12 | ++ | 0.9976 | 0.9905 |
| rs1333037 | | t | c | 0.5445 | 1.72 | 0.0767 | 1.29E-12 | ++ | 0.9964 | 0.9911 |
| rs1556515 | | t | c | 0.5445 | 1.72 | 0.0767 | 1.29E-12 | ++ | 0.9965 | 0.9917 |
| rs613312 | | a | g | -0.5577 | 0.57 | 0.0787 | 1.38E-12 | -- | 0.9821 | 0.9736 |
| rs1008878 | | t | g | 0.5434 | 1.72 | 0.0767 | 1.44E-12 | ++ | 0.9507 | 0.9821 |
| rs1412829 | | a | g | 0.5441 | 1.72 | 0.0773 | 1.99E-12 | ++ | 0.9997 | 0.9926 |
| rs679038 | | a | g | -0.5399 | 0.58 | 0.0775 | 3.25E-12 | -- | 0.9995 | 0.9972 |
| rs599452 | | a | g | -0.5399 | 0.58 | 0.0775 | 3.25E-12 | -- | 0.9997 | 0.9973 |
| rs543830 | | a | t | 0.5386 | 1.71 | 0.0775 | 3.64E-12 | ++ | 0.9965 | 0.9941 |
| rs1063192 | | a | g | 0.5191 | 1.68 | 0.0758 | 7.48E-12 | ++ | 0.9978 | 0.9999 |
| rs1360589 | | t | c | 0.5174 | 1.68 | 0.0762 | 1.14E-11 | ++ | 0.976 | 0.9677 |
| rs6475604 | | t | c | -0.5139 | 0.60 | 0.0761 | 1.45E-11 | -- | 0.9692 | 0.9656 |
| rs573687 | | a | g | -0.542 | 0.58 | 0.0805 | 1.70E-11 | -- | 0.9941 | 0.9955 |
| rs581876 | | t | c | -0.5389 | 0.58 | 0.0802 | 1.82E-11 | -- | 0.986 | 0.9887 |
| rs944800 | | a | g | -0.5579 | 0.57 | 0.0842 | 3.44E-11 | -- | 0.9187 | 0.9568 |
| rs10811648 | | t | c | 0.5091 | 1.66 | 0.0771 | 4.04E-11 | ++ | 0.9741 | 0.9702 |
| rs7030641 | | t | c | 0.4972 | 1.64 | 0.0757 | 5.09E-11 | ++ | 0.9836 | 0.9812 |
| rs7866783 | | a | g | -0.4965 | 0.61 | 0.0758 | 5.83E-11 | -- | 0.9853 | 0.9823 |
| chr9:22018406 | | a | c | -0.5543 | 0.57 | 0.086 | 1.13E-10 | -- | 0.9458 | 0.9922 |
| rs4977753 | | t | c | 0.4677 | 1.60 | 0.0726 | 1.16E-10 | ++ | 0.9924 | 0.9866 |
| rs944801 | | c | g | 0.4866 | 1.63 | 0.0757 | 1.27E-10 | ++ | 0.9644 | 0.9484 |
| rs10811651 | | a | g | 0.4954 | 1.64 | 0.0771 | 1.32E-10 | ++ | 0.9969 | 0.9949 |
| rs10965215 | | a | g | 0.4659 | 1.59 | 0.0725 | 1.34E-10 | ++ | 0.9956 | 0.9889 |
| rs10965224 | | a | t | 0.4951 | 1.64 | 0.0771 | 1.35E-10 | ++ | 0.9966 | 0.9969 |
| rs1333039 | | c | g | 0.4951 | 1.64 | 0.0771 | 1.35E-10 | ++ | 0.996 | 0.9967 |
| rs4977756 | | a | g | 0.4951 | 1.64 | 0.0771 | 1.35E-10 | ++ | 0.9985 | 0.9978 |
| rs1537378 | | a | g | -0.4951 | 0.61 | 0.0771 | 1.35E-10 | -- | 0.9941 | 0.9955 |
| rs2184061 | | a | c | 0.4951 | 1.64 | 0.0771 | 1.35E-10 | ++ | 0.9938 | 0.9944 |
| rs8181050 | | a | g | 0.4918 | 1.64 | 0.0767 | 1.42E-10 | ++ | 0.8654 | 0.865 |
| rs1333036 | | t | c | -0.4599 | 0.63 | 0.0725 | 2.24E-10 | -- | 0.9963 | 0.9884 |
| rs10115049 | | a | g | -0.4586 | 0.63 | 0.0723 | 2.30E-10 | -- | 0.9402 | 0.9142 |
| rs10811649 | | t | c | 0.4859 | 1.63 | 0.0769 | 2.68E-10 | ++ | 0.9453 | 0.945 |
| rs1591136 | | c | g | 0.4579 | 1.58 | 0.0725 | 2.72E-10 | ++ | 0.9981 | 0.9954 |
| rs7049105 | | a | g | -0.4579 | 0.63 | 0.0725 | 2.72E-10 | -- | 0.9988 | 0.9965 |
| rs10965212 | | a | t | 0.4579 | 1.58 | 0.0725 | 2.72E-10 | ++ | 0.996 | 0.992 |
| rs3217992 | | t | c | 0.4563 | 1.58 | 0.0724 | 2.90E-10 | ++ | 0.9973 | 0.9998 |
| rs2151280 | | a | g | 0.4535 | 1.57 | 0.0724 | 3.70E-10 | ++ | 0.9961 | 0.9934 |
| rs4977755 | | a | t | 0.4812 | 1.62 | 0.077 | 4.19E-10 | ++ | 0.9588 | 0.9536 |
| rs518394 | | c | g | -0.4715 | 0.62 | 0.0756 | 4.40E-10 | -- | 0.9575 | 0.9896 |
| rs523096 | | a | g | 0.4715 | 1.60 | 0.0756 | 4.40E-10 | ++ | 0.9575 | 0.9898 |
| rs2811713 | | a | g | -0.4977 | 0.61 | 0.0798 | 4.47E-10 | -- | 0.8998 | 0.9649 |
| rs615552 | | t | c | 0.4729 | 1.60 | 0.076 | 4.85E-10 | ++ | 0.9397 | 0.981 |
| rs1360590 | | t | c | -0.449 | 0.64 | 0.0723 | 5.42E-10 | -- | 0.9947 | 0.9833 |
| rs2383205 | | a | g | -0.4681 | 0.63 | 0.0761 | 7.52E-10 | -- | 0.8797 | 0.9131 |
| rs10757266 | | t | c | 0.4444 | 1.56 | 0.0724 | 8.16E-10 | ++ | 0.9727 | 0.9503 |
| rs10811645 | | a | g | 0.4444 | 1.56 | 0.0724 | 8.16E-10 | ++ | 0.9724 | 0.9502 |
| rs944799 | | a | g | -0.4444 | 0.64 | 0.0724 | 8.16E-10 | -- | 0.9718 | 0.9499 |
| rs10738605 | | c | g | -0.4444 | 0.64 | 0.0724 | 8.16E-10 | -- | 0.9752 | 0.9507 |
| rs10757265 | | t | c | -0.4444 | 0.64 | 0.0724 | 8.16E-10 | -- | 0.9755 | 0.9509 |
| rs7027950 | | t | c | 0.4444 | 1.56 | 0.0724 | 8.16E-10 | ++ | 0.9773 | 0.9513 |
| rs7028570 | | a | g | 0.4444 | 1.56 | 0.0724 | 8.16E-10 | ++ | 0.976 | 0.9511 |
| rs10120806 | | t | c | -0.4441 | 0.64 | 0.0724 | 8.38E-10 | -- | 0.978 | 0.9518 |
| rs10757267 | | c | g | 0.4439 | 1.56 | 0.0724 | 8.74E-10 | ++ | 0.9549 | 0.9517 |
| rs1412832 | | t | c | 0.5185 | 1.68 | 0.0846 | 9.00E-10 | ++ | 0.992 | 0.9891 |
| rs8181047 | | a | g | -0.5178 | 0.60 | 0.0846 | 9.16E-10 | -- | 0.9666 | 0.9818 |
| rs10120688 | | a | g | 0.4407 | 1.55 | 0.0724 | 1.17E-09 | ++ | 0.9934 | 0.9959 |
| rs2383204 | | a | g | -0.4407 | 0.64 | 0.0724 | 1.17E-09 | -- | 0.992 | 0.9943 |
| rs7028268 | | a | g | 0.4405 | 1.55 | 0.0725 | 1.25E-09 | ++ | 0.9649 | 0.9567 |
| rs7027048 | | a | g | -0.4386 | 0.64 | 0.0723 | 1.30E-09 | -- | 0.9545 | 0.9544 |
| rs2069418 | | c | g | 0.4561 | 1.58 | 0.0752 | 1.31E-09 | ++ | 0.9375 | 0.984 |
| rs1412830 | | t | c | -0.4704 | 0.62 | 0.079 | 2.66E-09 | -- | 0.8328 | 0.8697 |
| rs597816 | | t | c | 0.4614 | 1.59 | 0.0779 | 3.11E-09 | ++ | 0.9448 | 0.9867 |
| rs10757270 | a | g | -0.4166 | 0.66 | 0.071 | 4.35E-09 | -- | 0.9535 | 0.9462 |  |
| rs2069416 | a | t | 0.4223 | 1.53 | 0.0725 | 5.78E-09 | ++ | 0.9512 | 0.9895 |  |
| rs10811650 | a | g | -0.4129 | 0.66 | 0.071 | 6.07E-09 | -- | 0.9958 | 0.9932 |  |
| rs10811647 | c | g | -0.4129 | 0.66 | 0.071 | 6.07E-09 | -- | 0.9951 | 0.9942 |  |
| rs10738604 | a | g | 0.422 | 1.53 | 0.0728 | 6.64E-09 | ++ | 0.9384 | 0.9886 |  |
| rs10811641 | c | g | -0.4197 | 0.66 | 0.0726 | 7.35E-09 | -- | 0.9492 | 0.9873 |  |
| rs7035484 | c | g | -0.4135 | 0.66 | 0.0718 | 8.41E-09 | -- | 0.9587 | 0.9914 |  |
| rs567453 | c | g | -0.4248 | 0.65 | 0.0739 | 9.14E-09 | -- | 0.9833 | 0.9846 |  |
| rs9632885 | a | g | 0.4106 | 1.51 | 0.0715 | 9.49E-09 | ++ | 0.9616 | 0.9454 |  |
| chr9:21987015 | a | t | -0.5115 | 0.60 | 0.0893 | 1.02E-08 | -- | 0.896 | 0.9717 |  |
| rs643319 | a | c | -0.4216 | 0.66 | 0.0737 | 1.08E-08 | -- | 0.9808 | 0.985 |  |
| rs9632884 | c | g | 0.4083 | 1.50 | 0.0717 | 1.23E-08 | ++ | 0.9617 | 0.9456 |  |
| rs10965219 | a | g | -0.4109 | 0.66 | 0.0721 | 1.23E-08 | -- | 0.9038 | 0.9018 |  |
| rs496892 | t | c | -0.4199 | 0.66 | 0.0738 | 1.25E-08 | -- | 0.9843 | 0.9859 |  |
| rs504318 | a | t | 0.4199 | 1.52 | 0.0738 | 1.25E-08 | ++ | 0.9841 | 0.9857 |  |
| rs2106119 | a | g | -0.4066 | 0.67 | 0.0715 | 1.26E-08 | -- | 0.9567 | 0.9876 |  |
| rs2106120 | t | g | 0.4063 | 1.50 | 0.0714 | 1.29E-08 | ++ | 0.9567 | 0.9877 |  |
| rs10757263 | t | c | 0.4061 | 1.50 | 0.0714 | 1.31E-08 | ++ | 0.9566 | 0.988 |  |
| rs568447 | a | g | -0.4133 | 0.66 | 0.0728 | 1.38E-08 | -- | 0.929 | 0.9837 |  |
| rs10811640 | t | g | 0.4048 | 1.50 | 0.0714 | 1.43E-08 | ++ | 0.9566 | 0.9881 |  |
| rs10811643 | a | g | -0.4043 | 0.67 | 0.0717 | 1.70E-08 | -- | 0.9585 | 0.9909 |  |
| rs10811644 | a | t | -0.4043 | 0.67 | 0.0717 | 1.70E-08 | -- | 0.9586 | 0.9911 |  |
| rs10757269 | a | g | -0.4038 | 0.67 | 0.0717 | 1.80E-08 | -- | 0.962 | 0.9457 |  |
| rs7044859 | a | t | 0.3969 | 1.49 | 0.0714 | 2.67E-08 | ++ | 0.9566 | 0.9871 |  |
| rs10811652 | a | c | -0.392 | 0.68 | 0.0711 | 3.50E-08 | -- | 0.962 | 0.9564 |  |
