# Supplementary material for: Common Variants at 9p21 and 8q22 Are Associated with Increased Susceptibility to Optic Nerve Degeneration in Glaucoma
Source: PLoS Genet. 2012 Apr 26;8(4):e1002654. doi: 10.1371/journal.pgen.1002654 (PMC3343074; doi:10.1371/journal.pgen.1002654)
Supplement: Table S6 — CDKN2BAS haplotypes for the NEIGHBOR and GLAUGEN NPG datasets. Haplotype analysis of SNPs that were nominally significant in the CDKN2BAS regions in the NEIGHBOR and GLAUGEN NPG datasets. The haplotype with the most significant association is indicated in bold text. Abbreviations: Bp (base pair); Freq (frequency); NPG (normal pressure glaucoma); OR (odds ratio). (DOCX) [file pgen.1002654.s017.docx]

**Table S6. CDKN2BAS haplotypes for the NEIGHBOR and GLAUGEN NPG datasets**

|  |  | **GLAUGEN** | | | | | **NEIGHBOR** | | | | | rs3217992 | rs1063192 | rs573687 | rs7049105 | rs2157719 | rs2151280 | rs1412829 | rs10120688 | rs4977756 | rs1412832 |
| --- | --- | --- | --- | --- | --- | --- | --- | --- | --- | --- | --- | --- | --- | --- | --- | --- | --- | --- | --- | --- | --- |
| Bp - Start | Bp - End | Freq overall | Freq NPG | Freq Ctrl | OR | P | Freq overall | Freq NPG | Freq Ctrl | OR | P |  |  |  |  |  |  |  |  |  |  |
| 21993223 | 22067543 | 0.40 | 0.50 | 0.39 | 1.67 | 2.61E-06 | 0.41 | 0.54 | 0.39 | 1.53 | 1.04E-05 | A | A | G | G | A | A | A | A | A | A |
|  |  | 0.01 | 0.01 | 0.01 | 0.99 | 0.97 | 0.02 | 0.02 | 0.02 | 1.18 | 0.66 | G | A | G | A | A | G | A | A | A | A |
|  |  | 0.09 | 0.10 | 0.09 | 1.07 | 0.70 | 0.08 | 0.08 | 0.08 | 1.24 | 0.22 | G | A | G | A | A | G | A | G | A | A |
|  |  | 0.09 | 0.09 | 0.09 | 1.03 | 0.86 | 0.09 | 0.09 | 0.09 | 0.88 | 0.48 | G | A | G | G | A | A | A | A | A | A |
|  |  | 0.02 | 0.01 | 0.02 | 0.45 | 0.14 | 0.02 | 0.02 | 0.02 | 0.86 | 0.66 | G | G | A | A | G | G | G | G | A | A |
|  |  | 0.08 | 0.07 | 0.09 | 0.88 | 0.52 | 0.08 | 0.07 | 0.09 | 0.85 | 0.39 | G | G | A | A | G | G | G | G | G | A |
|  |  | **0.22** | **0.17** | **0.27** | **0.57** | **4.39E-05** | **0.23** | **0.15** | **0.27** | **0.54** | **5.09E-06** | **G** | **G** | **A** | **A** | **G** | **G** | **G** | **G** | **G** | **G** |
|  |  | 0.03 | 0.04 | 0.04 | 0.84 | 0.52 | 0.03 | 0.03 | 0.04 | 0.74 | 0.28 | G | G | G | A | G | G | G | G | G | G |
|  |  |  |  |  | Omnibus P | 1.06E-05 |  |  |  | Omnibus P | 1.91E-13 |  |  |  |  |  |  |  |  |  |  |

Haplotype analysis of SNPs that were nominally significant in the *CDKN2BAS* regions in the NEIGHBOR and GLAUGEN NPG datasets. The haplotype with the most significant association is indicated in bold text.
