# Supplementary material for: Common Variants at 9p21 and 8q22 Are Associated with Increased Susceptibility to Optic Nerve Degeneration in Glaucoma
Source: PLoS Genet. 2012 Apr 26;8(4):e1002654. doi: 10.1371/journal.pgen.1002654 (PMC3343074; doi:10.1371/journal.pgen.1002654)
Supplement: Table S7 — Haplotypes for 8q22 associated region for the NEIGHBOR and GLAUGEN NPG datasets. Haplotype analysis of SNPs that were nominally significant in the 8q22 region in the NEIGHBOR and GLAUGEN NPG datasets. Abbreviations: Bp, base pair; Freq, frequency; NPG, normal pressure glaucoma; OR, odds ratio. (DOCX) [file pgen.1002654.s018.docx]

**Table S7. Haplotypes for 8q22 associated region for the NEIGHBOR and GLAUGEN NPG datasets.**

|  |  | Neighbor | | | Glaugen | | |  |  |  |  |
| --- | --- | --- | --- | --- | --- | --- | --- | --- | --- | --- | --- |
| bp start | bp end | Freq | OR | P | Freq | OR | P | rs284489 | rs284494 | rs284495 | rs1521774 |
| 106027196 | 106048166 | 0.134 | 0.674 | 0.00895 | 0.145 | 0.592 | 0.000812 | G | G | G | G |
|  |  | 0.193 | 0.712 | 0.00815 | 0.198 | 0.677 | 0.00467 | G | A | G | G |
|  |  | 0.663 | 1.53 | 7.52E-05 | 0.649 | 1.64 | 1.00E-05 | A | A | A | A |
|  |  | omnibus P | | 0.02646 | omnibus P | | 6.72E-05 |  |  |  |  |

Haplotype analysis of SNPs that were nominally significant in the 8q22 region in the NEIGHBOR and GLAUGEN NPG datasets.
