# Supplementary material for: Common Variants at 9p21 and 8q22 Are Associated with Increased Susceptibility to Optic Nerve Degeneration in Glaucoma
Source: PLoS Genet. 2012 Apr 26;8(4):e1002654. doi: 10.1371/journal.pgen.1002654 (PMC3343074; doi:10.1371/journal.pgen.1002654)
Supplement: Table S8 — DNA sequence variants in LRP12 and ZFPM2 in NPG and POAG patients and controls. Abbreviations: Ref, reference allele; Obs, observed alleles; POAG, primary open angle glaucoma; NPG, normal pressure glaucoma, AA, amino acid; UTR-5, 5′ untranslated region; UTR-3, 3′ untranslated region; SYNON, synonymous. (DOCX) [file pgen.1002654.s019.docx]

**Table S8. DNA sequence variants in LRP12 and ZFPM2 in**

**NPG and POAG patients and controls**

| **LRP12** | | | | | | | | |
| --- | --- | --- | --- | --- | --- | --- | --- | --- |
| Exome analysis (50 POAG cases and 18 controls) | | | | | | | | |
| Position on chr 8 (bp) | dbSNP# | Ref | Obs | Effect | AA Change | Freq  Cases | Freq  Controls | dbSNP  av Het |
| 105502701 | NA | A | A/G | UTR-3 | NA | 0/50 | 1/18 | NA |
| 105503401 | rs16871494 | T | C/T | MISSENSE | SER,GLY | 0/50 | 2/18 | 0.076 |
| 105601151 | NA | G | A/G | UTR-5 | NA | 0/50 | 1/18 | NA |
| 105601162 | rs9694695 | G | A/G | UTR-5 | NA | 0/50 | 2/18 | 0.113 |
| 105601176 | rs9694676 | A | A/G | UTR-5 | NA | 0/50 | 4/18 | 0.113 |
| 105601192 | NA | A | A/C | UTR-5 | NA | 0/50 | 1/18 | NA |
|  | | | | | | | | |
| Resequencing 16 NPG cases | | | | | | | | |
| Position on Chr 8 (bp) | dbSNP# | Ref | Obs | Effect | AA Change | Freq  Cases | Freq  Controls | dbSNP  av Het |
| 105601162 | rs9694695 | G | A/G | UTR-5 | NA | 3/16 | NA | 0.113 |
| 105544297 | rs57833872 | C | C/T | INTRON | NA | 1/16 | NA | 0.365 |
| 105510326 | rs16871500 | T | G/T | INTRON | NA | 3/16 | NA | 0.242 |
|  | | | | | | | | |
| **ZFPM2** | | | | | | | | |
| Exome analysis (50 POAG cases and 18 controls) | | | | | | | | |
| Position on Chr 8 (bp) | dbSNP# | Ref | Obs | Effect | AA Change | Freq Cases | Freq Controls | dbSNP av Het |
| 106801042 | NA | G | G/C | MISSENSE | Ser/Thr | 0/50 | 1/18 | NA |
| 106813537 | NA | G | G/T | MISSENSE | Gln/His | 0/50 | 1/18 | NA |
| 106814279 | rs28374544 | A | A/G | MISSENSE | Ser/Gly | 0/50 | 2/18 | 0.114 |
| 106814656 | rs2920048 | G | G/C | MISSENSE | Glu/Asp | 2/50 | 1/18 | 0.078 |
| 106815474 | rs16873741 | C | C/T | MISSENSE | Ala/Val | 0/50 | 2/18 | 0.146 |
| 106815820 | rs16873745 | C | G/C | UTR-3 | NA | 0/50 | 2/18 | 0.144 |
| 106815856 | rs6991211 | C | G/G | UTR-3 | NA | 0/50 | 3/18 | 0.234 |
|  | | | | | | | | |
| Resequencing in 16 NPG cases | | | | | | | | |
| Position on Chr 8 (bp) | dbSNP# | Ref | Obs | Effect | AA Change | Freq  Cases | Freq  Controls | dbSNP  av Het |
| 106801171 | rs140283741 | G | G/A | INTRON | NA | 1/16 | NA | 0.039 |
| 106813518 | rs11993776 | C | C/G | MISSENSE | Ala/Gly | 1/16 | NA | 0.341 |
| 106814656 | rs2920048 | G | G/C | MISSENSE | Glu/Asp | 3/16 | NA | 0.078 |
| 106814695 | rs35998713 | C | C/G | SYNON | Val/Val | 2/16 | NA | 0.120 |
| 106815286 | rs1442320 | T | C/T | SYNON | Tyr/Tyr | 1/16 | NA | 0.104 |
| 106815517 | rs11995760 | C | C/T | SYNON | His/His | 1/16 | NA | 0.234 |

**Abbreviations: POAG, primary open angle glaucoma; NPG, normal pressure glaucoma, AA, amino acid; UTR-5, 5’ untranslated region; UTR-3, 3’ untranslated region; SYNON, synonymous.**
