# Supplementary material for: Common Variants at 9p21 and 8q22 Are Associated with Increased Susceptibility to Optic Nerve Degeneration in Glaucoma
Source: PLoS Genet. 2012 Apr 26;8(4):e1002654. doi: 10.1371/journal.pgen.1002654 (PMC3343074; doi:10.1371/journal.pgen.1002654)
Supplement: Text S1 — Supplemental Methods for exome sequencing and analysis. (DOCX) [file pgen.1002654.s020.docx]

**Common variants at 9p21 and 8q22 are associated with increased susceptibility to optic nerve degeneration in glaucoma**

Janey L Wiggs, Brian L Yaspan, Michael A Hauser, Jae Hee Kang, R Rand Allingham, Lana M. Olson, Wael Abdrabou, Baojian Fan, Danyi Wang, Wendy Brodeur, Donald L Budenz, Joseph Caprioli, Andrew Crenshaw, Kristy Crooks, Elizabeth DelBono, Kimberly F Doheny, David S Friedman, Douglas Gaasterland, Terry Gaasterland, Cathy Laurie, Richard Lee, Paul R Lichter, Stephanie Loomis, Yutao Liu, Felipe A Medeiros, Cathy McCarty, Daniel Mirel, Sayoko E Moroi, David C Musch, Anthony Realini, Frank W Rozsa, Joel S Schuman, Kathleen Scott, Kuldev Singh, Joshua D Stein, Edward H Trager, Paul VanVeldhuisen, Douglas Vollrath, Gadi Wollstein, Sachiko Yoneyama, Kang Zhang, Jason Ernst, Manolis Kellis, Robert N Weinreb, Tomohiro Masuda, Don Zack, Julia E Richards, Margaret Pericak-Vance, Louis R Pasquale, Jonathan L Haines

**Supplemental Methods:**

**Exome sequencing methods and analysis.**

**For each of 50 POAG and 18 random subjects, genomic DNA was sonicated into fragments of target length 250bp (Covaris) and prepared for paired-end sequencing (Illumina TruSeq Sample Prep Kit v2 (FC-121-2002), standard protocol). Each library was subjected to selection by hybridization with DNA probes designed to capture DNA from protein coding exons genome-wide (Nimblegen EZ-Exome Version 2.0 capture reactions, standard protocol), and sequenced (Illumina HiSeq 2000 instrument, TruSeq PE Cluster Kit v2.5 (PE-401-2510), TruSeq SBS Kit v2 (FC-401-3002), standard protocols) to generate paired sequence reads of length 100, *i.e.*, two 100 base reads per fragment. DNA samples for POAG cases were contributed by NEIGHBOR participants. Libraries were prepared at the High Throughput Sequencing Facility of The Scripps Research Institute, and sequencing was performed on instruments at Scripps and UCSD. Readpairs were mapped to a reference human genome (Version hg19, http://genome.ucsc.edu) using stringent mapping criteria (Bowtie mapping software, version 0.12.7; http://sourceforge.net/projects/bowtie-bio/files/bowtie/0.12.7/) [Langmead et al, 2009] as follows: Every readpair was required to match hg19 reference sequence with 97% identity and no gaps, with each read in the pair mapping within 1000 bases to accommodate a fragment length distribution and maximize data complexity; only readpairs that mapped to a unique site in the reference genome were retained. To eliminate PCR artifact, readpairs were flagged as duplicates if both reads shared mapping coordinates with another readpair, and only the best mapped pair was retained. Mapped reads were saved as sorted bam format files, and variants were determined from the read bases overlapping each reference genome position (Samtools version 0.1.12a,** [**http://samtools.sourceforge.net/**](http://samtools.sourceforge.net/)**) [Li et al, 2009). Variants were sorted by read-depth, with extremely high depth variants and variants with depth 1-4 flagged as suspect. Variants were annotated by gene structure location: amino acid codon, splice-site, intron, UTR or intergenic (CCDS release 9/7/2011 and RefSeq release 151); and codon-changing variants were assessed for impact on encoded proteins (SeattleSeq 134;** [**http://snp.gs.washington.edu/SeattleSeqAnnotation134/**](http://snp.gs.washington.edu/SeattleSeqAnnotation134/)**).**

**References:**

Langmead B, Trapnell C, Pop M, Salzberg SL. Ultrafast and memory-efficient alignment of short DNA sequences to the human genome. Genome Biol 10:R25, 2009.

Li H, Handsaker B, Wysoker A, Fennell T, Ruan J, Homer N, Marth G, Abecasis G, Durbin R and 1000 Genome Project Data Processing Subgroup (2009) The Sequence alignment/map (SAM) format and SAMtools. Bioinformatics, 25, 2078-2079.
